# Effect of Different Yeast Strains on Biogenic Amines, Volatile Compounds and Sensory Profile of Beer

**DOI:** 10.3390/foods11152317

**Published:** 2022-08-03

**Authors:** Mazvydas Matukas, Vytaute Starkute, Egle Zokaityte, Gintare Zokaityte, Dovile Klupsaite, Ernestas Mockus, João Miguel Rocha, Romas Ruibys, Elena Bartkiene

**Affiliations:** 1 Department of Food Safety and Quality, Veterinary Academy, Lithuanian University of Health Sciences, Tilzes Str. 18, LT-47181 Kaunas, Lithuania; mazvydas.matukas@stud.lsmu.lt (M.M.); vytaute.starkute@lsmuni.lt (V.S.); egle.zokaityte@lsmuni.lt (E.Z.); 2 Institute of Animal Rearing Technologies, Faculty of Animal Sciences, Lithuanian University of Health Sciences, Tilzes Str. 18, LT-47181 Kaunas, Lithuania; gintare.zokaityte@lsmuni.lt (G.Z.); dovile.klupsaite@lsmuni.lt (D.K.); ernestas.mockus@lsmuni.lt (E.M.); 3 Laboratory for Process Engineering, Environment, Biotechnology and Energy, Faculty of Engineering, University of Porto, 4200-465 Porto, Portugal; jmfrocha@fe.up.pt; 4 Associate Laboratory in Chemical Engineering, Faculty of Engineering, University of Porto, 4200-465 Porto, Portugal; 5 Institute of Agricultural and Food Sciences, Agriculture Academy, Vytautas Magnus University, K. Donelaicio Str. 58, LT-44244 Kaunas, Lithuania; romas.ruibys@vdu.lt

**Keywords:** yeast, beer, volatile compounds, biogenic amines, emotions induced for consumers

## Abstract

Nowadays, there are many sorts of beer, however, some of them, despite the good sensory and other quality indicators, could contain high concentrations of undesirable compounds, such biogenic amines (BA). The yeast strain (YS), used for fermentation, can cause desirable as well as undesirable changes in beer. The aim of this study was to evaluate the contribution of different YS (A-*Saccharomyces cerevisiae* var. *diastaticus*, B-*Saccharomyces cerevisiae* var. *bayanus*, C-*Brettanomyces claussenii*) on the main quality parameters of beer. In addition, the BA concentration and the volatile compounds (VC, measured by gas chromatography–mass spectrometry) and their relation with beer overall acceptability (OA, evaluated by 20 trained judges) and emotions induced for consumers were analysed. The YS was a significant factor on alcohol formation in beer (*p* = 0.0001). The highest colour intensity was shown by C beer (10.2 EBC), and the latter beer showed the lowest OA. All of the beer samples induced the highest intensity of the emotion “neutral”, and the main VC of the beer were 3-methyl-1-butanol; L-α-terpineol; hexanoic acid 3-methylbutyl ester; and n-capric acid isobutyl ester. The highest total BAs content was found in beer fermented with C. Finally, all of the tested YS are suitable for beer production, however, taking into consideration the safety aspect of the beer, it should be mentioned that the highest concentration of BAs was found in beer fermented with C strain.

## 1. Introduction

Beer can be derived from malted cereals or grains, along with water, hops and a yeast strain. The prepared wort is fermented with the selected yeast strain, and, during this process, fermentable sugars are converted into alcohol and carbon dioxide. Beer is a very popular beverage [1] and there are many beer sorts, as well as reported studies, about beer quality characteristics and the factors influencing the beer choice of consumers [2].

Although the raw materials used for beer preparation are similar, the secondary metabolites formed during the fermentation process depend on many factors (yeast strain used, temperature, pH, nutrients, among others), with the yeast strain used for fermentation being one of the most important factors. The brewing industry looks toward novel brewing starters to meet the consumer demand for increases in product assortment [3]. 

It has been reported that *Saccharomyces cerevisiae* var. *diastaticus* has amylolytic activity and, in most cases, causes undesirable changes in beer quality [4]. These changes are explained by its extracellular glucoamylase utilization of dextrins, which represent 10–20% of the total concentration of saccharides in the wort. However, the metabolism of dextrin and starch degradation is strain dependent, and it was reported that *S. cerevisiae* var. *diastaticus* yeast strains are suitable for brewing under similar conditions compared with classical brewing culture strains, resulting in desirable, appealing beers with clear taste [5]. Moreover, *S. cerevisiae* var. *diastaticus* can be used for a one-step process of starch fermentation to produce beers with low-carbohydrate content, as well as with specific sensory profile [5].

Although *S. cerevisiae* is the predominant yeast in most fermentation processes, other species of this genus have also been described to be involved in beverages fermentation [6]. 

The species *S. bayanus* is very complex, and at least two groups of *S. bayanus* strains have been reported [7,8,9]. The group *S. bayanus* var. *bayanus* includes the species contributing to the lager yeast genome, however, this group shows a very high degree of genetic variability [10]. 

Another yeast, *Brettanomyces*, is non-conventional and can be isolated from different sources [11]. In breweries, *Brettanomyces* are usually recognized as spoilage-causing microorganisms, because their presence can completely change the sensory characteristics of the beverage [12]. However, if applied correctly, *Brettanomyces* can produce metabolites that possess desirable exotic flavours [13]. Recently, the beer industry has broadened the application of *Brettanomyces* to create novel flavours in new beer style preparations [14]. The *Brettanomyces* species shows specific characteristics [14]; they produce limited amounts of glycerol, and can form a significant concentration of acetic acid [15]. In addition to glucose, most *Brettanomyces* strains can metabolize a variety of mono-, di- and trisaccharides, as well as dextrins [16]. *Brettanomyces* are suggested for the production of superattenuated and lower energy cereal beverages [17]. The range of amino acids *Brettanomyces* can use as nitrogen source is wide, glutamine being the most preferred one [18]. During the fermentation process, *Brettanomyces* produces a broad and complex spectrum of volatile compounds (VC) [19]. 

Taking into consideration that the different yeast strains could be used for various characteristics in beer preparation, in this study the same technological conditions and raw materials (except yeast strain) were applied for beer preparation and the influence of yeast strain on beer parameters was analysed. In addition to the most important beer characteristics, VC contribution to beer overall acceptability as well as emotions induced for consumers were evaluated. It was reported that food-evoked emotions have been argued to improve predictions regarding consumers’ food choices [20,21,22,23,24]. For this reason, we hypothesize, that the beer VC could contribute to emotions induced for consumers, as well as overall acceptability. In addition, biogenic amines (BAs) are often key flavour and aroma compounds critical to the character of fermented foods and beverages [25]. Furthermore, beer has been commonly reported, among foods and beverages, to be a health risk for some consumers, resulting from BAs intake [26]. The negative effects of BA on health derived from food have been reported, in which the BA content ranged from 301.8 to 500 mg/kg and from 190 to 500 mg/kg for tyramine and histamine, respectively [27,28,29,30,31,32,33,34]. Moreover, synergistic effects of separate BA can increase their adverse effects [35]. Most regulations focus on the most dangerous ones (histamine in certain foods such as fresh fish or enzyme-maturated fish products, allowing up to between 100 and 200 mg/kg, respectively) [36,37], and no regulation has been established for beer. Calculations of the dietary intake of BAs should consider all their potential sources from both foods and beverages. For this reason, control of the BAs in beer is very important, and we hypothesize that the formation of these compounds in beer could be dependent on yeast strain. 

The aim of this study was to evaluate the contribution of different yeast strains (*Saccharomyces cerevisiae* var. *diastaticus*, *Saccharomyces cerevisiae* var. *bayanus* and *Brettanomyces claussenii*) on the main quality parameters of beer (dry matter content, density, acidity parameters, real degree of fermentation (RDF), attenuation (ADF), energy value, ethanol and methanol concentrations, colour coordinates, bitterness units (BU)). In addition, the concentration of biogenic amines (BAs) and the profile of VC and their relationship with beer overall acceptability, as well as emotions induced for consumers, were analysed.

## 2. Materials and Methods

### 2.1. Materials Used for Beer Preparation

*Saccharomyces cerevisiae* var. *bayanus* yeast were purchased from ERBSLÖH Geisenheim GmbH (Geisenheim, Germany), *Saccharomyces cerevisiae* var. *diastaticus* yeast were obtained from Lallemand Inc. (Montréal, Canada) and *Brettanomyces claussenii* were bought from White Labs Inc. (San Diego, CA, USA). 

The hop varieties Citra brand, Amarillo brand and Mosaic brand were purchased from Yakima Chief Hops SA (Mont-Saint-Guibert, Belgium) and hop variety USA Sorachi Ace brand was bought from BarthHaas GmbH & Co KG (Nuremberg, Germany). 

Barley Pilsner, wheat and rye malts were obtained from JSC “Viking Malt” (Panevezys, Lithuania). 

The quality certificates for the raw materials are presented in Supplementary file 1. 

### 2.2. Beer Preparation 

**Figure 1:**
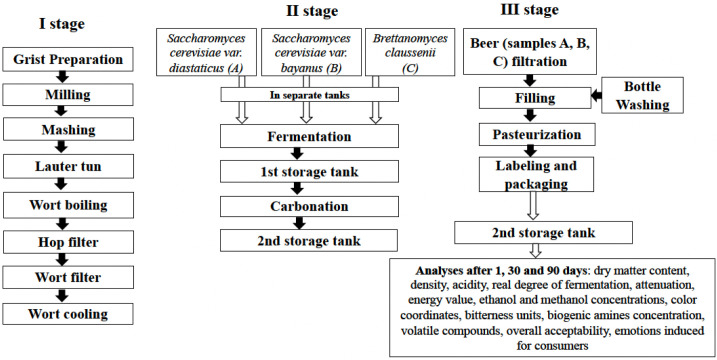
Principal technological scheme for the beer preparation.

The principal technological scheme for the beer preparation is given in Figure 1. 

All the malts were transferred into the grist mill (model Piccolo 11S, Sommer Maschinenbau GmbH, Elz, Germany) and grounded to mid-size parts. Then the malt grist was transferred to the brewhouse (model BH100, Plevnik, Dobrova, Slovenia) and mixed with water (mashed) (60 °C) in a mash conversion vessel (or mash tun), to give a soluble extract designed according to the recipe and activate the enzymes in the malts. Mashing involved increasing the temperature to the optimum temperature for the enzymes to act and maintaining the rest for a period of time as follows: 65 °C for 45 min for maltose production; 72 °C for 10 min for saccharification; 78 °C final mash temperature. At the end of the mashing process, the mash is transferred to a lauter tun vessel and lautering commences. Lautering is a filtration process in which the spent grain plays the role of the filter material. First, the wort draining occurs, then the spent grain is washed out with sparging hot water (80 °C). The obtained liquid is called wort and is boiled for 1 h. During this time the hops were added in three stages: 60 min for bitterness, 30 min for flavour and 1 min for aroma. 

After boiling, the wort is filtered, chilled and transferred to fermentation vessels (FUP 2 × 100, Plevnik, Dobrova, Slovenia). Three fermentation tanks were filled with the same wort to the same volume and temperature, then three different yeast strains (A, B, C) were pitched into each. The fermentation was carried out at 21–23 °C temperature. 

After fermentation, all three tanks were chilled to 4 °C, racked of the yeast and left for maturation.

After 0, 30 and 90 days of maturation, beer samples were subjected to analysis of the following parameters: dry matter content, density, pH, titratable acidity, real degree of fermentation (RDF), apparent degree of fermentation—attenuation (ADF), energy value, ethanol and methanol concentrations, colour intensity (EBC), bitterness units (BU), overall acceptability, emotions induced for consumers by beer, volatile compounds (VC) profile and biogenic amines (BA) concentration.

### 2.3. Evaluation of Beer Quality Parameters

Alcoholic grade (% *v*/*v*) was determined according to the standard method described in LST 1572 [38]. 

The dry matter, density, real degree of fermentation (RDF), apparent degree of fermentation (ADF), and energy value of beer samples were determined with an analyser DMA 4500 M (Anton Paar, Graz, Austria). 

The pH was measured using a pH electrode (PP-15; Sartorius, Goettingen, Germany), according to standard LST ISO 4316 [39]. 

The total acidity (TA, g/L) was determined according to standard LST 1990:2007 [40]. For the titration, a standard solution of sodium hydroxide (titrant) was used. A 50-mL volume of a decarbonated beer sample was pipetted into a 100-mL beaker. A magnetic stir bar was placed in the beaker and the pH probe immersed in the sample. While stirring the beer vigorously, beer was titrated with 0.1 M NaOH to pH 8.2 by adding in portions of about 1.5 mL up to pH 7.6, then in smaller increments of about 0.15 mL until a pH of 8.20 was reached. 

Colour intensity (EBC) was analysed spectrophotometrically according to standard LST 1490 [41]. 

The bitterness (BU) was determined with a LAMBDA™ 25 UV/Vis Spectrophotometer (PerkinElmer, Inc., Waltham, MA, USA) according to methodology described by Philpott et al. (1997) [42].

Overall acceptability testing of beer was carried out according to ISO method 8586-1 [43] by 20 trained judges for preliminary sensory acceptability using a 10-point Likert scale ranging from 10 (extremely like) to 1 (extremely dislike).

**Figure 2:**
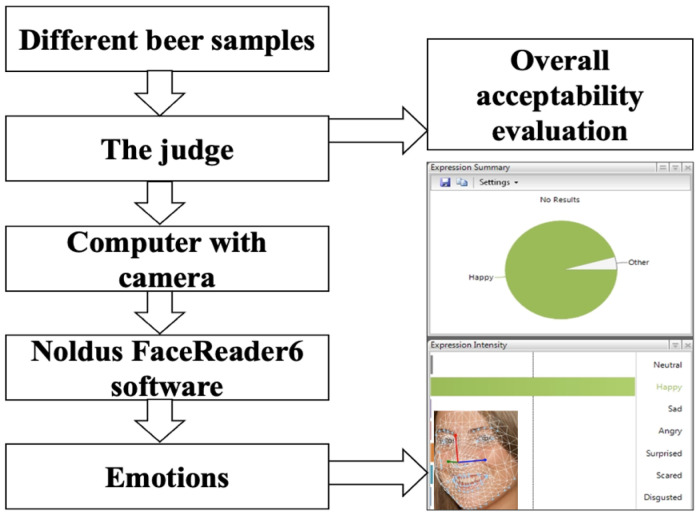
Analysis of the emotions induced by the beer using FaceReader 6 software (Noldus Information Technology, Wageningen, The Netherlands), and further scoring the eight emotion patterns: neutral, happy, sad, angry, surprised, scared, disgusted and contempt.

The beer samples were also tested (by the same 20 trained judges) by applying FaceReader 6.0 software (Noldus Information Technology, Wageningen, The Netherlands) (Figure 2), with a scoring scale of eight emotion patterns (neutral, happy, sad, angry, surprised, scared, disgusted, and contempt). The whole procedure is described in detail by Bartkiene et al. [44]. For statistical analysis, the maximum values of the facial expression patterns of the respective sections were used.

The VC of the beer samples were analysed by gas chromatography–mass spectrometry (GC-MS) as described by Bartkiene et al. [44], with some modifications which are described below. For headspace extraction, 2 g of degassed sample were used. The VC were identified according to mass spectrum libraries (NIST11, NIST11S, FFNSC2). 

The extraction and determination of BAs in beer samples followed the procedures developed by Ben-Gigirey et al. [45]. The BA separation was performed on Discovery ^®^ HS C18 column (150 × 4.6 mm, 5 μm; SupelcoTM Analytical, Bellefonte, Pennsylvania, USA). The BA were identified based on their retention times in comparison to their corresponding standards. Linearity of the calibration curves was established by injecting five concentrations of each biogenic amine standard (0.00–0.28 g/L for tryptamine, phenylethylamine, cadaverine, putrescine, histamine, tyramine, and spermidine; 0.01–1.40 g/L for spermine). Good linearity (*R*^2^: 0.9963–0.9999) was obtained between peak area and analyte concentration. 

### 2.4. Statistical Analysis

Statistical analysis was completed using IBM SPSS Statistics for Windows, v26 (IBM Corp., Armonk, NY, USA). The results were expressed as the mean values (for beer samples n = 3) ± standard error (SE). In order to evaluate the effects of different yeasts and different maturation duration on beer quality parameters, data were analysed by multivariate analysis of variance and Tukey HSD tests as post hoc tests. A linear Pearson’s correlation was used to quantify the strength of the relationship between the variables. The results were recognized as statistically significant at *p* ≤ 0.05.

## 3. Results and Discussion

### 3.1. Beer Samples’ Quality Parameters 

**Figure 3:**
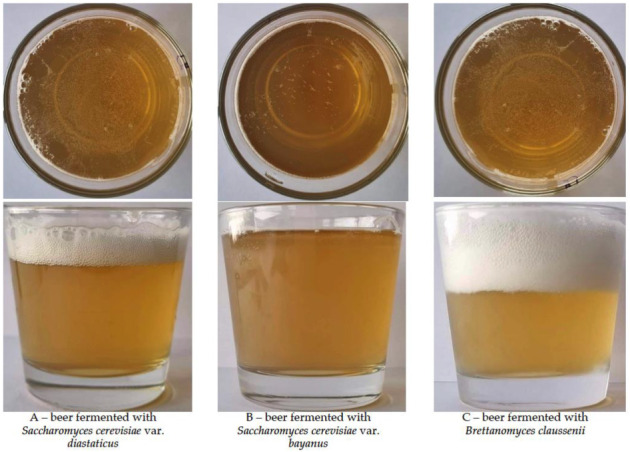
Pictures of the beer samples.

The main quality parameters of the samples are shown in Table 1 and beer pictures are given in Figure 3. Comparing the alcoholic grade during the different stages of maturation, no significant differences were found in the same sample groups, and, on average, in beer group A (fermented with *Saccharomyces cerevisiae* var. *diastaticus*) ethanol concentration was 7.92%, in group B (fermented with *Saccharomyces cerevisiae* var. *bayanus*)—6.55%, and in group C (fermented with *Brettanomyces claussenii*)—7.49%. In comparison different sample groups at the same maturation period, the highest alcoholic grade after 90 days of maturation was found in group A samples (8.14%), on average, 1.58% and 0.49% higher, in comparison with B and C groups, respectively. Multivariate analysis of variance showed that the yeast strain used for fermentation was a significant factor in beer alcoholic grade (*p* = 0.0001), but the duration of maturation and yeast strain * duration of maturation interaction did not have a significant effect on alcohol formation in beer. Comparing all the density parameters (Plato degrees, density, g/cm and SG), no significant differences were found between the groups, though, yeast strain * duration of maturation interaction did have a significant association with the Plato degree (*p* ≤ 0.001) of samples. The Plato degree shows the grams of dry extract per 100 g of wort, and 2 g of dry extract can produce, on average, 1 g of ethanol, theoretically [46]. Despite this, no correlation between the Plato degree and alcoholic grade of the samples was established. Furthermore, it should be pointed out that the alcohol tolerance of the yeasts is not a fixed characteristic, which could be related with yeast strain, nutrients, sugar availability in the substrate, etc. Despite not finding significant differences in the real degree of fermentation (RDF) in the same samples group during the different periods of maturation, in comparing the different samples groups, a significantly lower RDF was found (after 1, 30 and 90 days of maturation) in group B samples (lower, on average, by 12.50%, 15.04% and 16.88%, respectively). Moreover, no significant differences were found in the same group of samples in the apparent degree of fermentation (ADF), however, comparing different groups at the same maturation period, group B samples showed lower ADF in comparison with both, A and C groups, and group A samples showed the highest ADF after 90 days of maturation (98.85%). Regarding the samples’ pH, the lowest pH (during the whole period of maturation) was found in group C samples, and, significant differences between the A and B groups were not established. Notwithstanding, no correlations were found between the samples’ pH and titratable acidity (TA), and the highest TA, during the whole period, was found in the group C samples. Different tendencies of the colour intensity were established, and, after 90 days of maturation, the highest colour intensity was identified in group C samples (10.2 EBC). Between the colour intensity and beer samples’ density (both, in g/cm and SG) and TA significant correlations were established (r = 0.501, *p* = 0.008; r = 0.991, *p* = 0.0001; r = 0.581, *p* = 0.001, respectively). Colour is a very important characteristic of beer, related to its appearance [46]. Beer colour depends on melanoidins produced in the malting process, and, the type of malt is the main factor for colour formation [47]. In this study, the same raw materials were used for beer preparation, except yeast strain, therefore, it could be stated that the differences in colour were related to the different yeast strains used for fermentation. It has been reported that high fermentation yeasts produce beers with higher values of absorbance due to the browning and oxidation of the melanoidins [47]. Even so, as indicated above, in our case the technological parameters were the same, and colour was related to the yeast strain used for fermentation. Significant differences between the beer samples’ bitterness were not established and beer bitterness showed significant correlations with samples’ density (SG), TA and colour (r = 0.727, *p* = 0.0001; r = 0.617, *p* = 0.001; r = 0.765, *p* = 0.0001, respectively). The parameter used to classify beers according to their bitterness is the IBU (international bittering unit) [46]. Hops are the raw material in beer mainly responsible for beer bitterness [48]. The hops provide α-acids, which during the technological process are transformed into iso-α-acids; the latter are more bitter and, for this reason, the beer shows higher IBU values. Moreover, the addition of hops may simultaneously influence a beer’s bitterness, taste and aroma, and, this is a crucial factor, in that a beer which falls outside of the accepted range for the hop content may be judged as not belonging to the style it is categorized as [49]. By keeping the variety and quantity of hops constant across all beer samples, it could be stated that the yeast strain used for fermentation showed the same capacity to preserve the α-acids from hops, as differences in beer IBU were not found. 

No significant differences between the beer energy values were found, and on average, the beer energy value was 58 kCal/100 mL. Finally, analysed factors and their interaction were not significant on the samples’ RDF, ADF, pH, TA, colour intensity and energy value.

### 3.2. Overall Acceptability and Emotions Induced for Consumers by Beer Samples

Overall acceptability (OA) and emotions induced for consumers by the beer samples are given in Table 2. Comparing OA in the same group samples, no significant differences were found, but after 90 days of maturation, the lowest OA was shown by group C samples (on average, 5.2 points). OA of the beer showed significant positive correlation with the emotions “neutral” and “scared” (r = 0.518, *p* = 0.006 and r = 0.472, *p* = 0.013, respectively). Although the correlation between the OA and the emotion “happy” was not established, the emotion “happy” showed moderate negative correlation with the emotion “scared” (r = −0.606, *p* = 0.001). Regarding the intensity of the emotion “happy”, recorded for 90-day beer samples, the highest expression was achieved by group C samples, which showed the lowest OA. Overall, the highest expressed emotion was “neutral” for all the tested beer samples. Furthermore, the valence of most of the beer samples was negative, except samples A and C after 90 days and samples B after 1 day (0.007, 0.019 and 0.012, respectively). 

The results can be explained by the fact that beer is a traditional beverage, so the dominant “neutral” emotion could be explained by the judges being familiar with the tested beverage. Moreover, while bitterness is a characteristic by which beer is commonly characterized, it is not appreciated by all [50,51,52]. It has been reported that women usually prefer less bitterness and more complex flavours in beverages, in comparison with men [53,54]. Further, it was reported that individuals associate happiness and surprise with a sweet-tasting solution more often than with salty, sour or bitter solutions, whereas bitter solutions are associated with disgust [55]. The between-subject test showed that the analysed factors and their interaction were significant on most of the tested emotions induced for consumers, except “happy” (Table 3). Furthermore, the analysed factors were not significant on OA evaluated by the judges. Finally, it could be stated that the specific beer taste, especially bitterness, is associated with the expression of negative emotions, though the highest expression of “neutral” emotion leads to the conclusion that the specific beer taste is familiar to consumers.

### 3.3. Beer Volatile Compounds Profile 

The main volatile compounds (VC) of the beer samples are provided in Table 4, and the whole VC profile is given in Supplementary file 2 (Table S1. Beer volatile compounds profile). It was established that by increasing the duration of maturation, 3-methyl-1-butanol content in beer samples increased, with the highest content in group A beer samples after 90 days of maturation (20.56% of the total VC content). The compound 3-methyl-1-butanol gives a brandy, pleasant, fruity, and wine-like odour. This VC formation was significantly influenced by the duration of maturation (Table 5). Between 3-methyl-1-butanol content in beer samples and beer OA, a moderate positive correlation was established (r = 0.441, *p* = 0.021). Opposite tendencies of the styrene content in A and C beer group samples were found, and, after 90 days of maturation, its content in beer was reduced, on average, by 1.3 and 1.9 times, respectively. Styrene odour is described as sweet, balsam, floral, and plastic. Both the analysed factors and their interaction showed a significant influence on styrene formation in beer samples (Table 5). β-Myrcene content also showed a tendency to decrease during maturation: in group A samples by 1.6, in group B samples by 1.7, and in group C samples by 1.6 times. β-Myrcene content showed a moderate positive correlation with the intensity of the emotion “happy” (r = 0.575, *p* = 0.002) induced by beer samples. β-Myrcene formation was significantly influenced by duration of fermentation, and its odour is described as anise, grape, fruity, herbaceous, peach, sweet, vanilla, wine-like, vegetable, woody and green. Hexanoic acid, ethyl ester in beer samples ranged from 0.541% to 3.91% of the total VC content (in group B and C samples, respectively, after 90 days of maturation), and the yeast strain used for fermentation and the interaction of factors (yeast strain used for fermentation and duration of maturation) were significant for its formation. Hexanoic acid, ethyl ester odour is described as sour, fatty, sweat and cheese. The highest content of 2-methylbutylbutanoate in group B samples after 90 days of maturation was established (3.95% from the total VC content). The 2-methylbutylbutanoate content showed positive moderate correlation with beer OA (r = 0.401, *p* = 0.038). This VC odour is fruity, pear, apricot, apple, tropical, gooseberry, spicy and rummy, and, the yeast strain used for fermentation and duration of maturation interaction showed a significant influence on its formation. L-α-Terpineol was one of the main VC in the beer VC profile, but correlations with this VC content and OA as well as the emotion “happy” were not found. L-α-Terpineol odour is described as pine, terpene, lilac, citrus, woody and floral. Decanal, acetic acid, octyl ester and hexanoic acid 3-methylbutyl ester content in beer samples were lower than 2% of the total VC content, even so, a moderate positive correlation was established between the hexanoic acid 3-methylbutyl ester content in beer samples and OA of the beer (r = 0.403, *p* = 0.037). Decanal odour is sweet, aldehydic, waxy, orange, peel, citrus and floral; acetic acid, octyl ester odour is described as ethereal acetic, fruity, sweet, berry, and grape; hexanoic acid 3-methylbutyl ester odour is described as sour, fatty, sweat and cheese. All analysed factors and their interaction were significant on hexanoic acid 3-methylbutyl ester formation in beer. Hexanoic acid 2-methylbutyl ester was one of the main beer VC, whose content during the maturation process was slightly reduced in all the group samples, and, after 90 days of maturation, the highest content of this VC was found in group C samples (20.6% of the total VC content). Hexanoic acid 2-methylbutyl ester odour is described as sour, fatty, sweat and cheese, and, both factors: yeast strain used for fermentation and duration of maturation showed a significant influence on this VC formation (Table 5). Acetic acid, 2-phenylethyl ester content in group A and B samples showed a tendency to increase by increasing the duration of maturation, but in group C beer samples, an increase was established after just 30 days, then the acetic acid, 2-phenylethyl ester content started to reduce, and after 90 days of maturation this VC content was the lowest in group C samples, in comparison with group A and B samples. Acetic acid, 2-phenylethyl ester possesses honey, floral, sweet, rosy, green with fatty nuances, powdery, and slightly cinnamic odour. Despite the ethyl trans-4-decenoate content in the beer samples’ VC profile being lower than 2% of the total VC content, this VC showed a moderate negative correlation with samples’ OA (r = 0.459, *p* = 0.016). Ethyl trans-4-decenoate odour is described as green, fruity, waxy and cognac. Comparing the ethyl 9-decenoate (fruity and fatty odour) content in beer samples’ VC profile, after 90 days of maturation, the lowest content of this VC was found in group A samples (1.79% of the total VC content). Caryophyllene (spicy odour) was established in just four samples, in three of which after 90 days of maturation. α-Humulene was found only in group C samples (after 30 and 90 days of maturation), still a weak moderate correlation was found between this VC content and the emotion “happy” (r = 0.382, *p* = 0.049). α-Humulene odour is described as woody and slightly bitter. Both analysed factors and their interaction were significant on α-humulene formation in beer. In all the cases, during maturation, capric acid isobutyl ester (oily, sweet, brandy, apricot, fermented and cognac odour) content was reduced, in contrast to ethyl dodecanoate (sweet, clean, waxy, musk and animal odour), whose content showed increasing tendencies.

It was reported that beer fermented with *S. cerevisiae* var. *diastaticus* has a good sensory profile, and the capacity to build phenolic off-flavours is yeast strain-specific [5].

*Saccharomyces bayanus* belongs to the *Saccharomyces* genus and shows genetic similarity to other species that belong to this taxon, although they may vary in terms of oenological properties and the ability to produce volatile compounds [56]. It was reported that wines fermented with *S. bayanus* showed higher flavour intensity in comparison with *S. cerevisiae* [57]. The main differences being that *S. bayanus* synthesizes higher concentrations of 2-phenylethanol, ethyl lactate, 2-phenylethyl acetate and other acetate esters [57,58], while *S. cerevisiae* produces higher content of isobutanol, isoamyl alcohol and amyl alcohol [57].

It was reported, that the main differential of *Brettanomyces* strains is the production of volatile phenols, most notably 4-ethylphenol and 4-ethylguaiacol [14]. These compounds possess a diverse flavour comprising spicy, medicinal, leathery, horse sweat and smoky, among others [59,60]. Among the VC produced by *Brettanomyces*, esters are the most desired as they contribute a pleasant fruity flavour to beer, however, its formation is related to the *Brettanomyces* strain [14]. In addition, *Brettanomyces* shows the property to esterify middle- and long-chain fatty acids, which give a rancid and cheesy odour, into esters, switching the flavour profile towards sweet, grape, apple, and wine-like flavours [14].

Recent studies focusing on yeast strain selections are based on their improvement of ester production [61,62,63].

This study showed that the yeast strain is a significant factor in the main VC formation in beer, and despite some of the VC showing a low concentration in the main VC profile, they have a significant influence on beer OV.

### 3.4. Biogenic Amines Concentration in Beer Samples

Biogenic amines (BAs) concentration in beer samples is shown in Table 6. Histamine, tyramine, spermine and spermidine in beer samples were not established. In a comparison of the tryptamine (TRY) concentration, in groups A and C, after 30 days of maturation, the TRY concentration in beer samples increased, and in group B samples it remained similar to the first day of maturation. After 90 days of maturation, TRY in beer samples was not found. Opposite tendencies were found for phenylethylamine (PHE), and PHE concentration was increased by increasing the duration of maturation, and the highest concentration of PHE was found in group C samples after 90 days of maturation. Putrescine (PUT) was found in all beer samples after 90 days of maturation, as well as in group B and C beer samples after 30 days of maturation. Furthermore, the PUT concentration in group C samples after 30 and 90 days of maturation was, on average, 15 times higher than that in the group B samples. Cadaverine (CAD) was found only in group C samples after 30 and 90 days of maturation (223.8 and 182.5 mg/kg, respectively). The test of between-subjects effect (multivariate analysis of variance) showed that the yeast strain is a significant factor on PUTR and CAD concentration in beer samples (*p* ≤ 0.0001). The duration of maturation was a significant factor on all the identified BAs in beer: on TRY, PHE, PUTR and CAD content (*p* ≤ 0.0001, *p* = 0.008, *p* ≤ 0.0001, *p* ≤ 0.0001, respectively). Moreover, the yeast strain used for fermentation and maturation duration interaction were significant on TRY, PUTR and CAD content in beer samples (*p* = 0.009, *p* ≤ 0.0001, and *p* ≤ 0.0001, respectively). A very strong positive correlation was found between CAD and PUTR (r = 0.997, *p* ≤ 0.0001). Furthermore, between TRY and PHE, a moderate negative correlation was established (r = −0.432, *p* = 0.024).

It was reported, that in beer, the presence of BAs is mainly due to the activity of contaminants (mainly Enterobacteria sp.), which are possibly active at the early stage of the fermentation, and lactic acid bacteria (LAB) [64]. Moreover, some *S. cerevisiae* strains could produce PUTR, spermidine and spermine [64].

Correlations between BA concentration in beer samples and beer overall acceptability and emotions induced for consumers are shown in Table 7. In addition to BAs production, LAB can lead to the formation of organoleptic properties. This study showed, a weak positive correlation between the OA of beer and TRY (r = 0.382, *p* = 0.049). Between TRY and the emotion “happy” a negative moderate correlation was found (r = −0.552, *p* = 0.003) and between TRY and the emotion “surprised” a moderate positive correlation was established (r = 0.647, *p* = 0.0001). PHE, PUTR and CAD showed negative moderate correlations with the emotion “surprised” (r = −0.588, *p* = 0.001; r = −0.501, *p* = 0.008, and r = −0.459, *p* = 0.016, respectively). Furthermore, between the emotion “neutral” and PUTR and CAD negative moderate correlations were found (r = −0.515, *p* = 0.006, and r = −0.545, *p* = 0.003, respectively). The concentration of 100 mg/L or 100 mg/kg of BAs is considered a safe dose for most consumers, but in the case of alcoholic beverages this limit is much lower, because, the ethanol may reduce the detoxification mechanism of BAs [65]. Moreover, individuals who are taking MAO and DAO enzyme inhibitor drugs are in a higher risk group for the level of BAs in the diet [66]. It was reported that the main BAs in beer are: agmatine, histamine (HIS), CAD, PUTR, PHE, tyramine (TYR), spermine, spermidine and histamine [67,68,69]. Some BAs (spermine, spermidine, CAD and PUTR) do not affect health, but can form carcinogenic nitrosamines by reacting with nitrites. Furthermore, PUTR and CAD can increase the toxicity of other BAs [70,71,72]. 

The European legislation does not specify a BAs threshold, but the European Food Safety Authority (EFSA) has elaborated a scientific opinion on the risk associated with the formation of BAs in fermented products [73]. Based on the mean content in foods and consumer exposure data, fermented food categories were ranked with respect to histamine and tyramine, but the presently available information was insufficient to conduct quantitative risk assessment of BAs, individually and in combination(s). Further research on BAs in fermented foods is needed; particularly on the toxicity and acceptable concentrations, and production-process-based control measures. Beer has also been reported as a possible health risk for some consumers due to BAs intake [74]. Beer is considered as a source of the dietary polyamines, putrescine, spermidine and spermine [75]. Hypertensive crises in patients treated with drugs inhibiting monoamine oxidase after beer consumption have been reported in the literature [76,77,78,79]. Tyramine intake as low as 6 mg within a 4-h period or beers with tyramine content over 10 mg/L were considered as dangerous for consumption [79]. Finally, it is very important to control the concentration of BAs in beer, because, this beverage is very popular in many countries, and could be consumed in high amounts. Especially, taking into consideration that the most popular foods to consume with beer are cheese and some smoked meat products, this could lead to additional doses of the total BAs consumption.

## 4. Conclusions

The highest alcoholic grade was achieved in beer fermented with *Saccharomyces cerevisiae* var. *diastaticus* strain (8.14%), and the yeast strain used for fermentation was a significant factor on the alcohol concentration in beer (*p* = 0.0001). The highest colour intensity was shown by beer fermented with *Brettanomyces claussenii* (10.2 EBC), and the latter beer samples showed the lowest OA (5.2 points, on average). All beer samples induced the highest intensity of the emotion “neutral”, in comparison with other fixed emotions. The main VC of the beer samples were 3-methyl-1-butanol; styrene; β-myrcene; hexanoic acid, ethyl ester; 2-methylbutylbutanoate; L-α-terpineol; decanal; acetic acid, octyl ester and hexanoic acid 3-methylbutyl ester; hexanoic acid 2-methylbutyl ester; acetic acid, 2-phenylethyl ester; acetic acid, 2-phenylethyl ester; ethyl trans-4-decenoate; ethyl 9-decenoate; caryophyllene; α-humulene; capric acid isobutyl ester; and ethyl dodecanoate. In addition to VC, BAs also showed a tendency to influence beer OA and emotions induced for consumers. Histamine, tyramine, spermine and spermidine in beer samples were not established, but the highest total BAs content in beer fermented with *Brettanomyces claussenii* and the yeast strain was a significant factor on PUTR and CAD concentration in beer (*p* ≤ 0.0001). Finally, it could be stated that all of the tested yeast strains are suitable for beer production, however, concerning the safety aspect of the beer, it should be mentioned that the highest concentration of BAs was found in beer fermented with the *Brettanomyces claussenii* yeast strain.

## Figures and Tables

**Table 1:** The dry matter content, density, pH, real degree of fermentation (RDF), apparent degree of fermentation (ADF), energy value, ethanol and methanol concentrations, colour intensity, and bitterness of the beer samples (A—beer fermented with *Saccharomyces cerevisiae* var. *diastaticus*; B—beer fermented with *Saccharomyces cerevisiae* var. *bayanus*; C—beer fermented with *Brettanomyces claussenii*).

Parameters	Beer Samples								
	A			B			C		
	Duration of Maturation								
	1 Day	30 Days	90 Days	1 Day	30 Days	90 Days	1 Day	30 Days	90 Days
Alcoholic grade (% *v*/*v*)	7.67 ± 0.42 a,B	7.95 ± 0.36 a,B	8.14 ± 0.22 a,C	6.55 ± 0.34 a,A	6.55 ± 0.43 a,A	6.56 ± 0.27 a,A	7.32 ± 0.23 a,B	7.49 ± 0.34 a,B	7.65 ± 0.21 a,B
Dense materials, Plato %	15.30 ± 0.40 a,A	15.27 ± 0.24 a,A	15.29 ± 0.33 a,A	15.29 ± 0.35 a,A	15.28 ± 0.15 a,A	15.30 ± 0.20 a,A	15.30 ± 0.21 a,A	15.28 ± 0.32 a,A	15.26 ± 0.24 a,A
Density, g/cm	0.990 ± 0.007 a,A	0.990 ± 0.005 a,A	0.990 ± 0.006 a,A	0.988 ± 0.003 a,A	0.989 ± 0.005 a,A	0.986 ± 0.004 a,A	0.989 ± 0.005 a,A	0.989 ± 0.003 a,A	0.989 ± 0.002 a,A
Density, SG units	1.00 ± 0.08 a,A	1.00 ± 0.03 a,A	1.00 ± 0.07 a,A	1.01 ± 0.02 a,A	1.01 ± 0.03 a,A	1.01 ± 0.04 a,A	1.01 ± 0.05 a,A	1.01 ± 0.04 a,A	1.00 ± 0.06 a,A
RDF, %	76.66 ± 3.43 a,B	79.47 ± 2.67 a,B	81.29 ± 4.47 a,B	65.57 ± 2.75 a,A	65.58 ± 2.84 a,A	65.59 ± 3.85 a,A	73.22 ± 4.4 a,B	74.90 ± 3.23 a,B	76.52 ± 3.12 a,B
ADF, %	92.83 ± 4.91 a,B	96.48 ± 3.82 a,B	98.85 ± 2.68 a,C	78.58 ± 3.23 a,A	78.6 ± 3.36 a,A	78.61 ± 2.94 a,A	88.38 ± 2.84 a,B	90.55 ± 4.98 a,B	92.65 ± 2.57 a,B
pH	4.40 ± 0.03 a,B	4.42 ± 0.02 a,B	4.36 ± 0.04 a,B	4.44 ± 0.04 a,B	4.43 ± 0.03 a,B	4.40 ± 0.02 a,B	4.09 ± 0.02 a,A	4.10 ± 0.03 a,A	4.09 ± 0.01 a,A
TA (lactic acid), g/L	1.00 ± 0.010 c,B	0.800 ± 0.030 b,B	0.700 ± 0.040 a,B	0.400 ± 0.030 a,A	0.400 ± 0.020 a,A	0.400 ± 0.020 a,A	2.10 ± 0.110 c,C	1.80 ± 0.140 b,C	1.40 ± 0.130 a,C
Colour intensity, EBC	8.60 ± 0.07 a,A	10.3 ± 0.19 c,A	9.70 ± 0.07 b,B	10.2 ± 0.04 a,B	10.7 ± 0.05 b,C	10.2 ± 0.03 a,C	10.4 ± 0.04 b,C	10.6 ± 0.05 c,B	9.30 ± 0.06 a,A
Bitterness, BU	37.3 ± 2.80 a,A	35.8 ± 2.63 a,A	33.5 ± 1.93 a,A	34.2 ± 2.40 a,A	34.3 ± 2.71 a,A	34.1 ± 2.08 a,A	35.7 ± 3.16 a,A	38.4 ± 2.36 a,A	35.7 ± 3.49 a,A
Energy value, kCal/100 mL	58.0 ± 1.70 a,A	58.0 ± 2.50 a,A	58.0 ± 1.60 a,A	59.0 ± 2.60 a,A	59.0 ± 2.30 a,A	59.0 ± 1.80 a,A	58.0 ± 1.10 a,A	58.0 ± 1.40 a,A	58.0 ± 3.70 a,A
Energy value, kJ/100 mL	241 ± 3.6 a,A	240 ± 4.1 a,A	240 ± 5.7 a,A	243 ± 3.3 a,A	243 ± 2.4 a,A	243 ± 3.9 a,A	241 ± 2.1 a,A	241 ± 3.6 a,A	241 ± 2.7 a,A

Data expressed as mean values (n = 3) ± standard error (SE). a–c—mean values in line with beer samples fermented with the same yeast strain; A–C—mean values in line with beer samples fermented with a different yeast strain after the same duration of maturation; the means with different letters are significantly different (*p* ≤ 0.05). A—beer fermented with *Saccharomyces cerevisiae* var. *diastaticus*; B—beer fermented with *Saccharomyces cerevisiae* var. *bayanus*; C—beer fermented with *Brettanomyces claussenii;* RDF—real degree of fermentation; ADF—apparent degree of fermentation. SG units—specific gravity units; TA—titratable acidity.

**Table 2:** Overall acceptability and emotions induced for consumers by beer samples (A—beer fermented with *Saccharomyces cerevisiae* var. *diastaticus*; B—beer fermented with *Saccharomyces cerevisiae* var. *bayanus*; C—beer fermented with *Brettanomyces claussenii*).

	Beer Samples								
	A			B			C		
	Duration of Maturation								
	1 Day	30 Days	90 Days	1 Day	30 Days	90 Days	1 Day	30 Days	90 Days
	Overall acceptability from 10 (extremely like) to 1 (extremely dislike)								
	7.4 ±1.5 a,A	8.1 ± 2.3 a,A	8.0 ± 1.2 a,B	8.1 ± 2.0 a,A	8.4 ± 1.5 a,A	7.4 ± 1.0 a,B	6.9 ± 1.8 a,A	7.7 ± 2.4 a,A	5.2 ± 1.1 a,A
	Emotions induced by the beer (from 0 to 1)								
Neutral	0.691 ± 0.015 a,A	0.743 ± 0.016 b,A,B	0.783 ± 0.036 b,B	0.686 ± 0.042 a,A	0.784 ± 0.051 b,B	0.720 ± 0.029 b,A,B	0.725 ± 0.020 b,A	0.677 ± 0.012 a,A	0.676 ± 0.020 a,A
Happy	0.056 ± 0.010 b,B	0.034 ± 0.011 a,B	0.084 ± 0.009 c,B	0.098 ± 0.011 c,C	0.017 ± 0.002 a,A	0.060 ± 0.014 b,A	0.028 ± 0.004 a,A	0.049 ± 0.007 b,B	0.097 ± 0.013 c,B
Sad	0.053 ± 0.008 c,A	0.034 ± 0.006 b,A	0.022 ± 0.002 a,A	0.043 ± 0.004 b,A	0.033 ± 0.005 a,A	0.031 ± 0.003 a,B	0.055 ± 0.008 b,A	0.036 ± 0.003 a,A	0.052 ± 0.004 b,C
Angry	0.019 ± 0.003 a,A	0.035 ± 0.004 b,A	0.021 ± 0.002 a,A	0.030 ± 0.004 a,B	0.032 ± 0.005 a,A	0.057 ± 0.008 b,C	0.065 ± 0.009 b,C	0.039 ± 0.007 a,A	0.033 ± 0.004 a,B
Surprised	0.025 ± 0.003 c,B	0.013 ± 0.001 b,A	0.007 ± 0.002 a,A	0.019 ± 0.002 b,A	0.018 ± 0.002 b,B	0.005 ± 0.003 a,A	0.018 ± 0.002 c,A	0.010 ± 0.002 b,A	0.004 ± 0.003 a,A
Scared	0.016 ± 0.004 b,C	0.016 ± 0.004 b,B	0.004 ± 0.002 a,B	0.004 ± 0.001 a,A	0.013 ± 0.002 b,B	0.007 ± 0.003 a,B	0.008 ± 0.002 b,B	0.008 ± 0.001 b,A	0.002 ± 0.001 a,A
Disgusted	0.024 ± 0.005 b,B	0.014 ± 0.002 a,A	0.012 ± 0.003 a,A	0.024 ± 0.005 b,B	0.012 ± 0.003 a,A	0.009 ± 0.002 a,A	0.013 ± 0.002 a,A	0.021 ± 0.004 b,B	0.019 ± 0.005 b,A
Contempt	0.007 ± 0.003 a,A	0.004 ± 0.002 a,A	0.004 ± 0.002 a,A	0.011 ± 0.002 b,A	0.008 ± 0.002 b,A	0.004 ± 0.001 a,A	0.013 ± 0.002 b,A,B	0.004 ± 0.002 a,A	0.004 ± 0.001 a,A
	Valence (from −1 to 1)								
	−0.034 ± 0.007 b,B	−0.055 ± 0.009 a,A	0.007 ± 0.003 c,B	0.012 ± 0.006 c,C	−0.057 ± 0.007 a,A	−0.023 ± 0.008 b,A	−0.093 ± 0.012 a,A	−0.034 ± 0.006 b,B	0.019 ± 0.007 c,C

Data expressed as mean values (n = 20) ± standard error (SE). a–c—mean values in line with beer samples fermented with the same yeast strain; A–C—mean values in line with beer samples fermented with different yeast strains after the same duration of maturation; the means with different letters are significantly different (*p* ≤ 0.05). A—beer fermented with *Saccharomyces cerevisiae* var. *diastaticus*; B—beer fermented with *Saccharomyces cerevisiae* var. *bayanus*; C—beer fermented with *Brettanomyces claussenii.*

**Table 3:** Influence of the analysed factors and their interaction on overall acceptability and emotions induced for consumers by beer samples.

Factor	Dependent Variable	*p*
Yeast strain used for fermentation	OA	0.225
	neutral	0.0001
	happy	0.998
	sad	0.0001
	angry	0.0001
	surprised	0.0001
Duration of maturation	OA	0.377
	neutral	0.001
	happy	0.0001
	sad	0.0001
	angry	0.579
	surprised	0.0001
Yeast strain used for fermentation * Duration of maturation	OA	0.767
	neutral	0.0001
	happy	0.0001
	sad	0.0001
	angry	0.0001
	surprised	0.0001

*p*—significance, analysed factors and their interaction is significant, when *p* ≤ 0.05.

**Table 4:** The main volatile compounds of the beer samples (A—beer fermented with *Saccharomyces cerevisiae* var. *diastaticus*; B—beer fermented with *Saccharomyces cerevisiae* var. *bayanus*; C—beer fermented with *Brettanomyces claussenii*).

VC, % from the Total VC Content	Beer Samples									Correlations (r) and Their Significance (*p*)		
	A			B			C				between OA and VC	between ‘H’ and VC
	Duration of Maturation											
	1 Day	30 Days	90 Days	1 Day	30 Days	90 Days	1 Day	30 Days	90 Days			
3-methyl-1-butanol	10.52 a,B	18.0 b,B	20.56 b,B	14.41 a,C	15.69 a,A	21.67 b,B	8.47 a,A	14.95 b,A	17.11 b,A	r	0.441 *	0.317
										*p*	**0.021**	0.108
Styrene	4.26 a,B	6.13 a,B	5.64 a,B	9.25 a,B	11.89 a,C	11.97 a,C	1.93 b,A	3.28 c,A	0.992 a,A	r	0.131	0.067
										*p*	0.515	0.740
β-myrcene	1.14 b,A	1.06 b,A	0.718 a,A	1.43 b,A	1.38 b,A	0.820 a,B	1.47 b,A	1.42 b,A	0.911 a,C	r	−0.193	0.575 **
										*p*	0.336	**0.002**
Hexanoic acid, ethyl ester	1.10 a,A	1.41 a,B	1.26 a,B	2.33 a,B	3.08 b,C	3.91 b,C	0.931 b,A	0.599 a,A	0.541 a,A	r	−0.130	−0.197
										*p*	0.517	0.324
2-Methylbutylbutanoate	2.82 a,B	2.89 a,A	3.06 a,A,B	1.91 a,A	2.82 b,A	3.95 c,B	2.86 a,B	2.82 a,A	2.49 a,A	r	0.401 *	−0.228
										*p*	**0.038**	0.252
L-α-Terpineol	24.7 a,A	26.9 a,C	23.6 a,B	19.6 b,A	20.4 b,A	16.9 a,A	22.3 a,A	24.5 a,B	24.7 a,B	r	0.319	0.034
										*p*	0.104	0.867
Decanal	1.04 b,B	0.353 a,A	0.476 a,A	0.513 a,A	0.969 b,C	0.857 b,B	1.28 b,B	0.582 a,B	0.375 a,A	r	0.354	−0.068
										*p*	0.070	0.736
Acetic acid, octyl ester	0.682 b,A	0.430 a,A	0.357 a,A	0.750 b,B	0.607 b,B	0.357 a,A	0.986 c,C	0.682 b,B	0.504 a,B	r	0.178	−0.183
										*p*	0.373	0.360
Hexanoic acid 3-methylbutyl ester	0.702 a,B	0.705 a,B	0.803 b,B	0.411 a,A	0.377 a,A	0.355 a,A	1.81 b,C	0.796 a,B	1.28 b,C	r	0.403 *	0.052
										*p*	**0.037**	0.797
Hexanoic acid 2-methylbutyl ester	21.2 b,A	15.2 a,A	15.6 a,B	22.5 A	16.7 b,A	10.4 a,A	27.1 b,B	19.9 a,B	20.6 a,C	r	0.049	−0.297
										*p*	0.809	0.132
Acetic acid, 2-phenylethyl ester	3.59 a,B	4.75 b,B	4.53 b,B	2.32 a,A	2.88 a,A	4.76 b,B	3.21 a,B	4.05 b,B	3.38 a,A	r	0.259	−0.076
										*p*	0.191	0.706
Ethyl trans-4-decenoate	1.50 a,B	1.68 a,B	1.85 a,B	1.47 a,B	1.67 a,B	2.31 b,C	0.531 a,A	0.739 b,A	0.715 b,A	r	−0.459 *	0.131
										*p*	**0.016**	0.516
Ethyl 9-decenoate	1.21 a,A	1.70 b,A	1.79 b,A	1.84 a,B	2.24 a,B	2.08 a,B	1.64 a,A,B	2.25 b,B	2.31 b,B	r	0.101	0.223
										*p*	0.616	0.264
Caryophyllene	nd	0.375 a	0.644 b,A	nd	nd	0.740 A	nd	nd	1.06 B	r	0.184	0.171
										*p*	0.358	0.394
α-humulene	nd	nd	nd	nd	nd	nd	nd	1.48 a	2.43 b	r	−0.344	0.382 *
										*p*	0.079	**0.049**
n-Capric acid isobutyl ester	10.3 b,B	4.94 a,A	4.24 a,A	6.17 b,A	4.97 a,A	4.33 a,A	11.1 b,B	8.57 a,B	7.28 a,B	r	−0.073	−0.047
										*p*	0.717	0.815
Ethyl dodecanoate	5.91 a,C	7.75 b,C	8.76 c,C	3.89 a,B	4.71 b,A	6.21 c,B	2.91 a,A	3.95 b,A	4.75 c,A	r	0.308	0.167
										*p*	0.119	0.404

Data expressed as mean values (n = 5). nd—not determined; OA—overall acceptability; VC—volatile compound; ‘H’—emotion ‘happy’ detected by FaceReader. a–c—mean values in line with beer samples fermented with the same yeast strain; A–C—mean values in line with beer samples fermented with different yeast strains after the same duration of maturation; the means with different letters are significantly different (*p* ≤ 0.05). r—Pearson correlation coefficient; *p*—significance; *—correlation is significant at the 0.05 level (2-tailed); ** Correlation is significant at the 0.01 level (2-tailed). Significant correlations are marked in bold letters. A—beer fermented with *Saccharomyces cerevisiae* var. *diastaticus*; B—beer fermented with *Saccharomyces cerevisiae* var. *bayanus*; C—beer fermented with *Brettanomyces claussenii.*

**Table 5:** Influence of the analysed factors on beer volatile compounds formation.

Dependent Variable	Significance (*p*) of the Influence of Factor and Factors Interaction		
	Yeast Strain Used for Fermentation	Duration of Maturation	Yeast Strain Used for Fermentation * Duration of Maturation
3-methyl-1-butanol	0.156	**0.034**	0.539
Styrene	**0.001**	**0.0001**	**0.010**
β-Myrcene	0.256	**0.0001**	0.455
Hexanoic acid, ethyl ester	**0.0001**	0.107	**0.0001**
2-Methylbutylbutanoate	0.974	0.307	**0.023**
L-α-Terpineol	0.375	0.619	0.637
Decanal	**0.0001**	**0.0001**	0.062
Acetic acid, octyl ester	0.054	**0.007**	0.505
Hexanoic acid 3-methylbutyl ester	**0.0001**	**0.003**	**0.0001**
Hexanoic acid 2-methylbutyl ester	**0.002**	**0.0001**	0.337
Acetic acid, 2-phenylethyl ester	**0.010**	0.152	0.886
Ethyl trans-4-decenoate	**0.0001**	0.089	0.722
Ethyl 9-decenoate	**0.0001**	**0.009**	**0.003**
Caryophyllene	**0.029**	**0.001**	0.093
α-humulene	**0.0001**	**0.0001**	**0.0001**
n-Capric acid isobutyl ester	**0.0001**	**0.0001**	**0.047**
Ethyl dodecanoate	**0.0001**	**0.0001**	0.121

Influence of factor and factors interaction is significant when *p* ≤ 0.05. Significant correlations are marked in bold letters.

**Table 6:** Biogenic amines concentration of the beer samples (A—beer fermented with *Saccharomyces cerevisiae* var. *diastaticus*; B—beer fermented with *Saccharomyces cerevisiae* var. *bayanus*; C—beer fermented with *Brettanomyces claussenii*).

Biogenic Amines Concentration, mg/kg	Beer Samples								
	A			B			C		
	Duration of Maturation								
	1 Day	30 Days	90 Days	1 Day	30 Days	90 Days	1 Day	30 Days	90 Days
Tryptamine	42.95 ± 5.63 a,A	75.34 ± 8.62 b,A	nd	60.56 ± 9.37 a,B	66.46 ± 7.03 a,A	nd	60.92 ± 7.21 a,B	69.51 ± 5.63 b,A	nd
Phenylethylamine	18.51 ± 1.29 a,A	24.46 ± 3.41 b,A	35.37 ± 3.72 c,A	22.99 ± 4.54 a,A,B	30.54 ± 2.15 b,B	36.95 ± 5.02 b,A	23.13 ± 2.07 a,A	29.31 ± 1.29 b,A,B	57.73 ± 5.41 c, B
Putrescine	nd	nd	17.58 ± 2.9 A	nd	22.27 ± 2.51 a,A	20.12 ± 2.13 a,A	nd	348.93 ± 9.78 b,B	305.1 ± 8.52 a,B
Cadaverine	nd	nd	nd	nd	nd	nd	nd	223.8 ± 14.2 b	182.5 ± 11.3 a
Histamine	nd	nd	nd	nd	nd	nd	nd	nd	nd
Tyramine	nd	nd	nd	nd	nd	nd	nd	nd	nd
Spermidine	nd	nd	nd	nd	nd	nd	nd	nd	nd
Spermine	nd	nd	nd	nd	nd	nd	nd	nd	nd

Data expressed as mean values (n = 3) ± standard error (SE); nd—not determined. a–c—mean values in line with beer samples fermented with the same yeast strain; A–C—mean values in line with beer samples fermented with different yeast strains after the same duration of maturation; the means with different letters are significantly different (*p* ≤ 0.05). A—beer fermented with *Saccharomyces cerevisiae* var. *diastaticus*; B—beer fermented with *Saccharomyces cerevisiae* var. *bayanus*; C—beer fermented with *Brettanomyces claussenii.*

**Table 7:** Correlation between biogenic amines concentration in beer samples and beer overall acceptability and emotions induced for consumers.

	Pearson Correlation and Significance	Tryptamine	Phenylethylamine	Putrescine	Cadaverine
Overall acceptability	Pearson Correlation	0.382 *	0.028	−0.269	−0.252
	Sig. (2-tailed)	0.049	0.890	0.175	0.205
Emotion “neutral”	Pearson Correlation	0.021	0.151	−0.515 **	−0.545 **
	Sig. (2-tailed)	0.916	0.453	0.006	0.003
Emotion “happy”	Pearson Correlation	−0.552 **	0.361	0.241	0.233
	Sig. (2-tailed)	0.003	0.065	0.226	0.242
Emotion “sad”	Pearson Correlation	0.205	−0.264	0.124	0.156
	Sig. (2-tailed)	0.304	0.183	0.538	0.439
Emotion “angry”	Pearson Correlation	0.100	0.025	−0.015	−0.008
	Sig. (2-tailed)	0.620	0.903	0.940	0.967
Emotion “surprised”	Pearson Correlation	0.647 **	−0.588 **	−0.501 **	−0.459 *
	Sig. (2-tailed)	0.0001	0.001	0.008	0.016

** Correlation is significant at the 0.01 level (2-tailed). * Correlation is significant at the 0.05 level (2-tailed).

## Data Availability

The data are available from the corresponding author, upon reasonable request.

## Supplementary Materials

The following supporting information can be downloaded at: https://www.mdpi.com/article/10.3390/foods11152317/s1. Supplementary file 1. Characteristics of the raw materials; Supplementary file 2. Table S1. Beer volatile compounds profile.
