# Supplementary material for: Effect of Different Yeast Strains on Biogenic Amines, Volatile Compounds and Sensory Profile of Beer
Source: Foods. 2022 Aug 3;11(15):2317. doi: 10.3390/foods11152317 (PMC9368685; doi:10.3390/foods11152317)
Supplement: Supplementary file 1 [file foods-11-02317-s001.zip › Supplementary file 1. Characteristics of the raw materials.pdf]

25/08

306 Division Street
Yakima, Wa 98902
Phone 800-952-4873
www.yakimachief.com

YAKIMA CHIEF HOPS

Certificate of Analysis

Supplier: **Yakima Chief Hops**
306 Division Street
Yakima, WA 98902

Date of Issue: 14-Aug-2020

2021 06 16
160 kg
15 Ulenos
Šarž: AA902965037
Krov. v: 4900266422/2021

Product Name: **Type 90 Hop Pellets**

Lot Number: P91-DUMOS4263
Variety: Mosaic® Brand, HBC 369
Crop Year: 2019
Production Date: December 2019
Best Use Before Date: December 2022

Characteristic	Typical Analysis	Actual Analysis
**Alpha Acids:**	2.5 - 17.5% (w/w)<br>HPLC by EBC Analytica V 7.7	11.4%
**Beta Acids:**	3.0 - 9.0% (w/w)<br>HPLC by EBC Analytica V 7.7	3.0%
**Cohumulone:**	As Percentage of Total Alpha Acids<br>HPLC by EBC Analytica V 7.7	20.0%
**Hop Storage Index:**	Varies by Variety and Time from Harvest<br>Hop Storage Index by ASBC Hops - 12	0.269
**Moisture:**	ASBC HOPS - 4C	9.8%
**Pesticides Residues:**	Comply with US Regulations and EC Directive 396/2005 Amendments	

**Lead:**	< 1.0 ppm	passes test
**Arsenic:**	< 0.5 ppm	passes test
**Cadmium:**	< 0.03 ppm	passes test
**Total Heavy Metals:**	< 10 ppm (asPb)	passes test

Missy Raver
Missy Raver
Quality Control Manager

BarthHaas®

ANALYSIS REPORT & CERTIFICATE OF CONFORMANCE

Our contract No.:	V90263
Your reference No:	4501046650/4500225348
Consignee:	Svyturys Brewery<br>Kuliu Vartu 7<br>91250 KLAIPEDA<br>LITAUEN
Type of product:	Hop Pellets Type 90
Variety:	USA Sorachi Ace
Crop:	2020
Batch No.:	I-00517-1
Quantity:	10 kgs net
Marks:	PELLETS TYPE 90<br>USA SORACHI ACE / CROP 2020<br>BATCH I-00517-1 / 2 x 5 KG<br>ALPHA 10,8 % ASBC<br>PROD DATE: 03/2021 / V90263

2021 07 13
10 kg
PO 4500225348
Dok.Nr 5000017424
Krov. v.: LS00784871
Sciauniai ski
03/2024

ANALYTICAL FIGURES:
(as is)

Alpha Acids:	10,8 %
**Method of analysis:**	ASBC spectro
**Country of origin:**	USA

We herewith confirm that the a-m goods conform to the respective legislation of the country of origin and the country of delivery.

The product conforms to the Carlsberg Specifications of 02.01.2018.

Nürnberg, 14.07.2021

For specification, material safety data sheets and details on quality control guarantee please see **www.barthhaas.com**

BarthHaas GmbH & Co. KG · Freiligrathstr. 7-9 · 90482 Nuremberg · Germany · Phone: +49 911 5489-0 · Fax: +49 911 5489-330 · info@barthhaas.de · www.barthhaas.com

15/02

YAKIMA CHIEF HOPS™

Yakima Chief Hops SA
45, rue des Sablières/Unit 66
1435 Mont-Saint-Guibert
Belgium

Phone: +32 (0)10 47 50 50
www.yakimachief.com

VAT BE 0462 003 476

YAKIMA CHIEF HOPS

Certificate of Analysis

Supplier: **Yakima Chief Hops SA**
45, rue des Sablières/Unit 66
1435 Mont-Saint-Guibert
Belgium

Date of Issue: 05-Feb-2021

2021 07 26
60 kg = 462 kg
PO 7000 [illegible]
Dok Nr: 50000 [illegible]

Product Name: **Type 90 Hop Pellets**

Lot Number: P91-EUVG15080
Variety: Amarillo® Brand, VGXP01, Unite
Crop Year: 2020
Production Date: November 2020
Best Use Before Date: November 2023

Characteristic	Typical Analysis	Actual Analysis
**Alpha Acids:**	2.5 - 17.5% (w/w)<br>LCV by EBC Analytica V 7.5	7.7%
**Oil by Distillation:**	Varies by Variety<br>ASBC Hops - 13 (%v/w)	1.0%

Oil Profile by GC, EBC Analytica V 7.12:

**B-Pinene:**	0.6 %	**Farnesene:**	8.3%
**Myrcene:**	35.2 %	**Humulene:**	24.3%
**Linalool:**	0.7 %	**Geraniol:**	0.3%
**Caryophyllene:**	8.8 %		

**Lead:**	< 1.0 ppm	passes test
**Arsenic:**	< 0.5 ppm	passes test
**Cadmium:**	< 0.03 ppm	passes test
**Total Heavy Metals:**	< 10 ppm (asPb)	passes test

Missy Raver
Missy Raver
Quality Control Manager

YAKIMA CHIEF HOPS™

Yakima Chief Hops SA
45, rue des Sablières/Unit 66
1435 Mont-Saint-Guibert
Belgium

Phone: +32 (0)10 47 50 50
www.yakimachief.com

VAT BE 0462 003 476

YAKIMA CHIEF HOPS

Certificate of Analysis

Supplier: **Yakima Chief Hops SA**
45, rue des Sablières/Unit 66
1435 Mont-Saint-Guibert
Belgium

Date of Issue: 08-Sep-2021

2021 0913
200 kg
PO 4500227291
Dol. Nr: 5000028189

Product Name: **Type 90 Hop Pellets**

Lot Number: P92-EUCIT0356
Variety: Citra® Brand, HBC 394
Crop Year: 2020
Production Date: January 2021
Best Use Before Date: January 2024

Characteristic	Typical Analysis	Actual Analysis
**Alpha Acids:**	2.5 - 17.5% (w/w)<br>LCV by EBC Analytica V 7.5	13.0%
**Oil by Distillation:**	Varies by Variety<br>ASBC Hops - 13 (%v/w)	1.7%

Oil Profile by GC, EBC Analytica V 7.12:

**B-Pinene:**	0.7 %	**Farnesene:**	0.1%
**Myrcene:**	55.5 %	**Humulene:**	12.6%
**Linalool:**	0.7 %	**Geraniol:**	0.3%
**Caryophyllene:**	7.8 %		

**Lead:**	< 1.0 ppm	passes test
**Arsenic:**	< 0.5 ppm	passes test
**Cadmium:**	< 0.03 ppm	passes test
**Total Heavy Metals:**	< 10 ppm (asPb)	passes test

Missy Raver
Missy Raver
VP of Quality

VIKING MALT

Certificate of Analysis

Product number	Lot number		Supplier	Supplier name
310001	0701046646		100977	Viking Malt Sp z o o
**Product**				
Viking Rye Malt 25 kg bag	Rugių salyklas		2021 12 06	
**Blending date**	**Best before date**	**Print date**	300 kg	
23.07.2021	23.07.2023	08.12.2021		
**Country of orig**	**Sample ID**		PO 4500230595	
PL	00053191		Dok Nr 5000029812	

Test descr	U/M	Result	MU%	Min	Max	Meas mtd	Lab
Color of Malt	°EBC	6.8		4.0	10.0	EBC 4.7.2	VM LAB
Extract of Malt, fine grind	% dw	85.5		81.0		EBC 4.5.1	VM LAB
Moisture	%	4.7			6.0	EBC 4.2	VM LAB

This document is generated by a traceable ERP system and therefore has no signature.

Postal address
Viking Malt UAB
Pramonės g 2

LT-35100 Panevėžys Lithuania

Domicile
Panevėžys

VAT no. LT107769917

Telephone
+370 45 468811

UAB „Grainmore"
Plačioji gatve 27, LT - 21146
Senųjų Trakų k. Trakų raj. Lietuva

2021 12 08
750 kg

GRAINMORE

Kokybės pažymėjimas Nr.00941
2021-12- 06

**Gamintojas: UAB "Grainmore"**	
**Įmonės/asmens kodas: 303074973**	
**Adresas: Plačioji g. 27, LT-21146, Senieji Trakai, Lietuva**	
**PRODUKTAS**	**Dribsniai avižiniai AD180, 25 kg**
**Užsakymo numeris**	**450023592**

Partijos nr.	**Kiekis, kg**	**Geriausias iki**
PR41203_1	750	03.12.2022

**TECHNINĖS SAVYBĖS**	
Techninė vertė	Skaitinė vertė
Drėgmės kiekis, ne daugiau kaip, %	12.0
Rūgštingumas, ne daugiau kaip, mg KOH g/100 g	90
Peleningumas, ne daugiau kaip, %	2.1
Aruodiniai kenkėjai	neleidžiama
Storis, mm	0.7-0.9
Peroksidazės aktyvumas	Neigiamas
Kvapas/spalva	Būdinga atitinkamiems dribsniams

**MAISTINGUMO DEKLARACIJA (100 g)**	
Energinė vertė (kcal/ kJ)	378/1593
Riebalai (g)	7,6
- iš kurių: sočiųjų riebalų rūgščių (g)	1,42
Angliavandeniai (g)	59,4
- iš kurių: cukrų (g)	1,3
Skaidulinės medžiagos (g)	9,1
Baltymai (g)	13,5
Druska (NaCl) (g)	0,02

**PAKAVIMAS**					
Polipropileno maišai	☐ 10kg	☒ 25kg	☐ 40kg	☐ 50kg	☐ big bags
Medžiagos, kurios liečiasi su maistu atitinka EB reglamentą 1935/2004 ir ES reglamentą 10/2011	☒ Yes		☐ No		
Žymėjimas atitinka ES reglamentą 1169/2011	☒ Yes		☐ No		

**SAUGOJIMAS, TRANSPORTAVIMAS IR GALIOJIMO LAIKAS**	
Laikymo ir transportavimo sąlygos	Prekių saugojimo patalpos temperatūra nuo +5 °C iki +25 °C, santykinis drėgnumas ne didesnis nei 75 %. Prekių saugojimo patalpos turi būti sausos, švarios, gerai vėdinamos, apsaugotos nuo tiesioginių saulės spindulių, neužkrėstos grūdų ir miltų kenkėjais. Kartu draudžiama laikyti medžiagas, skleidžiančias dulkes, specifinius kvapus
Galiojimo laikas	12 mėnesių

Pažymėjimą išdavė:
Laboratorijos vadovė Renata Žemaitienė
Laboratorijos vadovė

PO 4500230592
Dok Nr: 5000029545

Įmonės kodas: 303074973
PVM kodas: LT100007784713

+370 37 719657
info@grainmore.com
www.grainmore.com

VIKING MALT

Certificate of Analysis

minėrų salyklas

2021 07 27
24020 kg
PO 4500225841
GL 500004 5925

Product number	Lot number	
301001	P00601031366	
**Product**		
Pilsner Malt bulk		
**Blending date**	**Best before date**	**Print date**
19.07.2021	19.07.2023	19.07.2021
**Country of orig**	**Sample ID**	
LT	00035181	

Test descr	U/M	Result	MU%	Min	Max	Meas mtd	Lab
Betaglucan Content of Malt, calc	mg/l	82			200	EBC 4.16.2	VM LAB
Boiled Wort Colour, calc	°EBC	5.5					VM LAB
Color of Malt, calc	°EBC	4.0			4.0	EBC 4.7.2	VM LAB
Diastatic Power of Malt, calc	WK dw	263.00		250.00		EBC 4.12.1	VM LAB
Extract of Malt, fine grind, calc	% dw	81.5		81.0		EBC 4.5.1	VM LAB
Extract of Malt, fine grind, as calc	% dw	77.80				EBC 4.5.2A	VM LAB
Filtration time, fine calc	min	28					VM LAB
Friability, calc	%	86		80		EBC 4.15	VM LAB
Glassy kernels, calc	%	1.4			2.0	EBC 4.15	VM LAB
PUG, calc	%	3.9			4.0	EBC 4.15	VM LAB
Gushing, water	g	0			0	Carls. Mod	VM PAN
Moisture, calc	%	4.6			5.0	EBC 4.2	VM LAB
pH of Wort, calc		6.08		5.80		EBC 8.17	VM LAB
Protein, calc	%	10.70		9.00	11.50	EBC 4.3.1	VM LAB
Saccharification time, calc		<10		10		EBC 4.5.1	VM LAB
Soluble Nitrogen of Malt, calc	mg/100g	645.00		560.00	720.00	EBC 4.9.1	VM LAB

20% Planet, crop 2020
20% Avalon, crop 2020
60% Laureate, crop 2020

This document is generated by a traceable ERP system and therefore has no signature.

Postal address
Viking Malt UAB
Pramonės g 2

LT-35100 Panevėžys Lithuania

Domicile
Panevėžys

VAT no. LT107769917

Telephone
+370 45 468811

VIKING MALT

Certificate of Analysis

2021 07 14
1010 kg
Dok Nr: S0000172228
Kiek.v: 363 92

**Product number**	**Lot number**	
308004	P00601018545	
**Product**		
Viking Wheat Malt 750 kg big bag		
**Blending date**	**Best before date**	**Print date**
02.03.2021	02.03.2023	14.07.2021
**Country of orig**	**Sample ID**	
LT	00020484	

Test descr	U/M	Result	MU%	Min	Max	Meas mtd	Lab
Boiled Wort Colour, calc	°EBC	7.3					VM LAB
Color of Malt, calc	°EBC	5.0		3.0	5.0	EBC 4.7.2	VM LAB
Diastatic Power of Malt, calc	WK dw	275.00		275.00		EBC 4.12.1	VM LAB
Extract Difference, calc	% dw	1.00		1.00	1.60	EBC 4.5.2A	VM LAB
Extract of Malt, fine grind, calc	% dw	85.2		84.0		EBC 4.5.1	VM LAB
Extract of Malt, fine grind, as calc	% dw	81.30				EBC 4.5.2A	VM LAB
Filtration time, fine calc	min	55			60		VM LAB
Gushing, water	g	0			0	Carls. Mod	VM PAN
Moisture, calc	%	4.7			5.5	EBC 4.2	VM LAB
pH of Wort, calc		6.06		5.60	6.10	EBC 8.17	VM LAB
Protein, calc	%	10.90			13.00	EBC 4.3.1	VM LAB
Sieving test 2,2 - 2,5 mm, malt	%	1.9			2.0	EBC 3.11.1	VM LAB
Foreign matter, malt	%	0.4			1.0	EBC 3.11.1	VM LAB
Sieving test >2,5 mm, malt	%	94.4		93.0		EBC 3.11.1	VM LAB
Soluble Nitrogen of Malt, calc	mg/100g	756.00		700.00	1100.00	EBC 4.9.1	VM LAB
Viscosity of Wort, calc	mPa·s	1.95			1.85	EBC 8.4	VM LAB

100% Dekan, crop 2020

This document is generated by a traceable ERP system and therefore has no signature.

Postal address
Viking Malt UAB
Pramonės g 2

LT-35100 Panevėžys Lithuania

Domicile
Panevėžys

VAT no. LT107769917

Telephone
+370 45 468811

1 Bezeichnung des Stoffes / der Zubereitung und des Unternehmens Identification of the substance/preparation and of the company/undertaking

1.1 Bezeichnung des Stoffes oder der Zubereitung / Identification of the substance or preparation
Dry selected yeast.

**Artikelnummer / Art.-no.**	**Handelsname / Trade name**
3010x	Oenoferm® Belle Arome F3
3011x	Oenoferm® Freddo F3
3012x	Oenoferm® Müller-Thurgau F3
3013x	Oenoferm® Riesling F3
3014x	Oenoferm® Terra F3
3015x	Oenoferm® PinoType F3
3016x	Oenoferm® Tipico F3
3017x	Oenoferm® Bouquet F3
3018x	Oenoferm® InterDry F3
3019x	Oenoferm® Klosterneuburg F3
3020x	Oenoferm® F3
3021x	Oenoferm® Structure F3
3022x	Oenoferm® Rouge F3
3023x	Oenoferm® Color F3
3024x	Oenoferm® Rosé F3
3026x	Oenoferm® Zweigelt F3
3027x	Oenoferm® Veltiner F3
3040x	Oenoferm® X-treme
3041x	Oenoferm® wild & pure
3044x	Oenoferm® Trollinger
3045x	Oenoferm® X-Thiol

1.2 Verwendung des Stoffes / der Zubereitung / Use of the substance / preparation
1.2.1 Identifizierte Verwendung(en) / Identified use(s)
Industrie / Gewerbe / Industry / Professional: According to Erbslöh product leaflet.
Privat / Private: None.

1.3 EG-Inverkehrbringer / EC market responsible person
1.3.1 Bezeichnung des Unternehmens / Company/undertaking identification
Erbslöh Geisenheim AG, Erbslöhstraße 1, 65366 Geisenheim, Germany
E-Mail: info@erbsloeh.com, Internet: www.erbsloeh.com

1.3.2 Notrufnummer / Emergency telephone:
Tel: +49 (0) 6722 708-0: Diese Nummer ist nur zu Bürozeiten von Mo-Fr besetzt. This number is serviced during office hours from Monday-Friday.

2 Mögliche Gefahren / Hazards identification

2.1 Einstufung / Classification:
The preparation is not dangerous in the sense of EU legislation.

2.2 Andere Gefahren / Other hazard(s):
No special remarkable hazards for human and environment.

3 Zusammensetzung/Angaben zu Bestandteilen / Composition/information on ingredients

3.1. Chemische Charakterisierung (Stoff) / Chemical characterization (substance)
Chemische Bezeichnung / Chemical name: : Dry selected yeast: *Saccharomyces cerevisiae*
Synonym(e) / Synonyme(s): ---.
Gefahrensymbol(e) / Hazard(ous) symbol(s):
H-Sätze / H-phrases: None.
Gefährliche Bestandteile / Verunreinigungen / Hazard(ous) ingredients / impurity(ies):
None.
3.2. Zusätzliche Hinweise / Additional information:
No additional information.

4 Erste-Hilfe-Maßnahmen / First aid measures

4.1 Allgemeine Hinweise / General informations
No special measures are necessary.
4.2 Nach Einatmen / In case of inhalation
Provide for fresh air.
4.3 Nach Hautkontakt / In case of skin contact
Wash off with water and soap.
4.4 Nach Augenkontakt In case of eye contact
In case of contact with eyes, rinse immediately thoroughly with plenty of water and consult an opthalmologist.
4.5 Nach Verschlucken / In case of ingestion
Rinse mouth immediately and drink plenty of water. Call in physician.
4.6 Selbstschutz des Ersthelfers / Self-protection of the first aider
No special measures are necessary.

5 Maßnahmen zur Brandbekämpfung / Fire-fighting measures

5.1 Geeignete Löschmittel / Suitable extinguishing media
Co-ordinate fire-fighting measures to the fire surroundings.
5.2 Löschmittel, die aus Sicherheitsgründen nicht zu verwenden sind / Extinguishing media which must not be used for safety reasons
Co-ordinate fire-fighting measures to the fire surroundings.
5.3 Besondere Gefährdungen / Special exposure hazards
No special hazards.
5.4 Besondere Schutzausrüstung für die Brandbekämpfung / Special protective equipment for fire-fighters
Co-ordinate protective clothing to the fire surroundings.

6 Maßnahmen bei unbeabsichtigter Freisetzung / Accidental release measures

6.1 Personenbezogene Vorsichtsmaßnahmen / Personal precautions
Avoid generation of dust. Risk of slipping by spilt material in case of contact with water.
6.2 Umweltschutzmaßnahmen / Environmental precautions
No special environmental measures are necessary.
6.3 Verfahren zur Reinigung / Methods for cleaning up
Take up mechanically. Rinse with plenty of water.
6.4 Zusätzliche Hinweise / Additional information
No additional information.

7 Handhabung und Lagerung / Handling and Storage

7.1. Handhabung / Handling
Working places should be designed allow cleaning at any time. Avoid generation of dust.
7.2. Lagerung / Lagerbedingung / Storage / storage conditions
Store in a cool, dry, well-ventilated place, protected from foreign odours and vapours. Reseal opened packages tightly. Observe general requirements for the storage of foodstuffs.
7.3. Bestimmte Verwendung(en) / Specific use(s)
See product leaflet.

8 Begrenzung und Überwachung der Exposition / persönliche Schutzausrüstung / Exposure controls / Personal protection

8.1. Expositionsgrenzwerte / Exposure limit values
No substance related limit value available.
8.2. Begrenzung und Überwachung der Exposition / Exposure controls
8.2.1. Begrenzung und Überwachung der Exposition am Arbeitsplatz / Occupational exposure controls.
a) Atemschutz / Occupational exposure controls
Wear dust protection mask P2.
b) Handschutz / Hand protection
Suitable gloves are recommended.
c) Augenschutz / Eye protection
Safety glasses are recommended.
Andere Schutzmaßnahmen / Additional protection measures:
When using do not eat, drink, smoke, sneeze. Cleaning with water and soap.

8.2.2. Begrenzung und Überwachung der Umweltexposition / Environmental exposure controls
See section 7. No additional measures necessary.
8.2.3. Begrenzung und Überwachung des Stoffes in Verbraucher- oder Folgeprodukten / Substance control in consumer or downstream products
No special measures necessary.

9 Physikalische und chemische Eigenschaften / Physical and chemical Properties

9.1 Erscheinungsbild / Appearance

**Form / Physical state:**	Granulate.
**Farbe / Colour:**	Beige to bright brown.
**Geruch / Smell:**	Typical.

9.2 Wichtige Angaben zum Gesundheits- und Umweltschutz sowie zur Sicherheit / Important health, safety and environmental information

**Schüttgewicht / Bulk density**:	600-750 g/L.
**Dichte / density**:	Not applicable.
**pH-Wert (20 °C) / pH-value:**	No data available.
**Schmelzpunkt / Melting point :**	Not applicable.
**Siedepunkt / Boiling temperature :**	No data available.
**Selbstentzündlichkeit / Autoignition :**:	No self-ignition.
**Explosionsgefahr / Explosive properties:**	Dust explosible.
**Wasserlöslichkeit / Water solubility:**	Suspensible.

10 Stabilität und Reaktivität / Stability and Reactivity

10.1 Zu vermeidende Bedingungen / Conditions to avoid
Danger of dust explosion. Avoid electrostatic charge. Thermal decomposition takes place at temperatures above 100 °C.
10.2 Zu vermeidende Stoffe / Materials to avoid
Ammonia release in case of contact with strong acid or alkaline solution.
10.3 Gefährliche Zersetzungsprodukte / Hazardous decomposition products
None – in case of appropriate handling.

11 Toxikologische Angaben / Toxicological information

11.1 Toxokinetik, Stoffwechsel und Verteilung / Toxicocinetics, metabolism and distribution
According to the present state of knowledge toxic effects are not expected.
11.2 Akute Wirkungen (akute Toxizität, Reiz- und Ätzwirkung) / Acute Effects (acute toxicity, Irritant and corrosive effects)
According to the present state of knowledge acute toxic effects are not expected.

12 Umweltbezogene Angaben / Ecological information

12.1 Ökotoxizität / Ecotoxicity
According to the present state of knowledge negative ecological effects are not expected.
12.2 Mobilität / Mobility
According to the present state of knowledge negative ecological effects are not expected.
12.3 Persistenz und Abbaubarkeit / Persistence and degradability Product is biodegradable.
12.4 Bioakumulationspotential / Bioaccumulative potential No indication to bioaccumulation potential.
12.5 Ergebnis der Ermittlung der PBT-Eigenschaften / Results of PBT assessment
This substance does not meet the criteria for classification as PBT or vPvB.
12.6 Andere schädliche Wirkungen / Other adverse effects
The product may lead to a high biological consumption of oxygen in biological sewage plants or natural waters, which can have a negative effect on living beings.

13 Hinweise zur Entsorgung / Disposal considerations

13.1 Entsorgung / Abfall (Produkt) / Appropriate disposal / product
Dispose of waste according to applicable local, state, and federal regulations.
Empfehlung / Recommondation
None.
13.2 Entsorgung der Verpackung / Appropriate disposal / packaging
Dispose of waste according to applicable local, state, and federal regulations.

14 Angaben zum Transport / Transport information

14.1 Landtransport (ADR/RID/GGVSE) / Land transport (ADR/RID)
No dangerous good in the sense of this transport regulation.
14.2 See (IMDG), Binnen (A.D.N.R.) / Seeschiffstransport (IMDG), Binnenschiffstransport (ADNR)
No dangerous good in the sense of this transport regulation..
14.3 Luft (IATA) / Air transport (IATA)
No dangerous good in the sense of this transport regulation.

15 Rechtsvorschriften / Regulatory information

15.1 EU-Vorschriften / EU-Regulations
Gemäß GHS - Verordnung (EG) 1272/2008 / according to GHS - regulation (EC) 1272/2008
Gefahrensymbole und Gefahrenbezeichnung / hazard symbol(s) and labelling of the product
Not required.
Gefahrbestimmende Komponente(n) zu Etikettierung / Hazard(ous) component(s) for labelling:
Not applicable.
H- Sätze / H-phrases
Not required.
P- Sätze / P-phrases
Not required.
15.2 Stoffsicherheitsbeurteilung / Chemical Safety Assessment
For this product a chemical safety assessment was not carried out.
Besondere Kennzeichnung bestimmter Zubereitungen / Special provisions concerning the labelling of certain preparations
Please adhere to EU legislation.
15.3 Nationale Vorschriften / National regulations
Wassergefährdungsklasse / Water hazard class (WGK): nwg (non-water polluting substance).

16 Sonstige Angaben / Other information

16.1 Nummer und Wortlaut der H-Sätze unter Abschnitt 3 / Relevant H-phrases under section 3 (Number and full text):
None.
16.2 Schulungshinweise Training instructions: None.
16.3 Weitere Informationen / Further information: Please refer to our internet website for more information..

Mit den vorstehenden Angaben, die dem heutigen Stand unserer Kenntnisse und Erfahrungen entsprechen, wollen wir unser Produkt im Hinblick auf etwaige Sicherheitserfordernisse beschreiben, verbinden damit jedoch keine Eigenschaftszusicherungen und Qualitätsbeschreibungen. Bestehende Gesetze und Bestimmungen sind vom Anwender in eigener Verantwortung zu beachten.
With the information given, which complies with our present knowledge and experience, we would like to describe our products in view of possible safety requirements, however we do not combine any warranty of product property or quality. Laws and regulations currently in force, have to be adhered to by the user.

NON-ORGANIC PRODUCT DECLARATION

USDA National Organic Program Compliance of Non-Organic Ingredient or Processing Aid

Product: White Labs Brewer's Yeast (Saccharomyces Cerevisiae)

Manufacturer: White Labs Inc.

Indicate whether the three statements for "All Products" are True or False. Then find the type of product that applies (citric acid, color, flavor, etc.), and indicate whether the statements are True or False for that product type.

The product indicated above conforms to the following criteria:

	True	False
☐ All Products		
1. No genetically engineered micro-organisms were used in production.	X	
2. This product has not been irradiated.	X	
3. Sewage sludge was not used in production of the product or ingredients.	X	
☐ Citric Acid		
• Produced by microbial fermentation of carbohydrate substance.		
☐ Colors		
• Derived from non-synthetic* source(s) only.		
☐ Flavors		
• Derived from non-synthetic* source(s) only.		
• No synthetic solvents or carrier systems were used in production.		
• Does not contain any artificial preservative(s).		
☐ Enzymes		
• Derived from edible, nontoxic plants, nonpathogenic fungi, or nonpathogenic bacteria.		
☒ Yeasts		
• This yeast is non-synthetic*.	X	
• No growth on petrochemical substrate and/or sulfite waste liquor.	X	
• Not available in an organic form.	X	

The above statements regarding the above-named product produced by this company are true to the best of my knowledge, and are based on information provided by our supplier or vendor.

Name (please print): Bryan Espiritu

Title/Department: Quality Assurance Technician

Signature: Bryan Espiritu

Date: 03/01/2016

*Nonsynthetic (natural). A substance that is derived from mineral, plant, or animal matter and does not undergo a synthetic process as defined in section 6502(21) of the Act (7 U.S.C. 6502(21)). For the purposes of this part, nonsynthetic is used as a synonym for natural as the term is used in the Act.

Synthetic. A substance that is formulated or manufactured by a chemical process or by a process that chemically changes a substance extracted from naturally occurring plant, animal, or mineral sources, except that such term shall not apply to substances created by naturally occurring biological processes.

LALLEMAND INC.

January 23, 2020

Declaration for LalBrew® "Active Dry Yeast" for use in Brewing Practices

Product of Denmark: Produced by Danstar Ferment AG, Vejlevej 10 Fredericia DK-7000 Denmark |Subsidiary of Lallemand Inc.
Product of Austria: Produced by Lallemand GmbH, Ottakringerstrasse 89, Vienna, A-1160, Austria |Subsidiary of Lallemand Inc.

Food Safety

These products have been processed in a manner consistent with current Good Manufacturing Practices and HACCP. Practices include traceability, non-conformance, and recall. The facilities have written, implemented, recorded, and reviewed plans for manufacturing, processing, packaging, and holding food items. Additionally each production and storage facility has a plan specific to their building to address facility security and food security.

Under the aforementioned manufacturing conditions these products are safe for their intended use and suitable for consumption.

Food Allergens

The product(s) sold have not been produced with the foods or their derivatives that account for the majority of human food allergic reactions as listed in EU Reg. 1169/2011 as amended.

These products are not considered to contain the following:

- Cereals containing GLUTEN and products thereof
- Crustaceans and products thereof
- Eggs and products thereof
- Fish and products thereof
- Peanuts and products thereof
- Soy and products thereof
- Milk (including lactose) and products thereof
- Tree Nuts and products thereof
- Celery and products thereof
- Mustard and products thereof
- Sesame seeds and products thereof
- Lupine and products thereof
- Molluscs and product thereof
- Sulphur dioxide and sulphites at concentrations of more than 10 mg/kg or 10 mg/litre expressed as SO2

Food Contact Packaging

We hereby confirm that the packaging materials of these products are suitable for the packaging of food. We have certification on file from our suppliers documenting the packaging provided to us is in accordance with the current US and EU food contact packaging regulations.

Genetic Modification

info@lallemand.com | www.lallemand.com
1620 Préfontaine | Montréal | H1W 2N8 | Canada | Tel: +1 866 773 4246 | Fax: +1 514 221 3063

According to our knowledge, the microorganisms are as they were found in nature and have not been modified through genetic engineering. They have been rigorously checked and analyzed for identity and purity.

To the best of our knowledge materials used to make the final products do not contain GMO. We have documentation on file from suppliers as confirmation.

In these conditions, the aforementioned food materials do not contain GMO.

Radioactivity and Ionization
The products have not been ionized or irradiated and do not contain any ionized or irradiated components. The ingredients used in the fermentation and in the final product have not been irradiated or contain any ionized components as affirmed by our ingredient suppliers.

Use of Sewage Sludge
Sewage sludge has not been used in the production of the microorganisms.

Sewage sludge has not been used in the production of the ingredients contained in the aforementioned products as affirmed by our suppliers.

Growth on Petrochemical Substrate
The yeast products have not been grown on petrochemical substrate or sulphate waste liquor.

Ingredients of Animal Origin
The aforementioned microorganisms were not produced by or with ingredients of animal origin. In addition the finished products were not produced by or with ingredients of animal origin.

These products are therefore free of any risk from BSE (Bovine Spongiform Encephalopathy) and TSE (Transmissible Spongiform Encephalopathy).

Phthalates
Phthalates or its derivatives are not used in the production of the microorganisms or the ingredients used in the final products.

We have on file certification from our packaging suppliers that state that phthalates or its derivatives [bis-phenol A, and poly brominated substances (PBBs & PBDEs)] will not be added or be present in any package or packaging component during the manufacturing process. Or they provide the list of substances used, which does not include any phthalates or its derivatives

Dioxins and PCB
There is no limit requirement for these products or their ingredients on dioxin levels under Commission Regulation (EC) No 1881/2006 "Setting maximum levels for certain contaminants in foodstuffs" section 5. Therefore these products or products made with these ingredients do not constitute a risk for Dioxin and PCB compounds.

info@lallemand.com | www.lallemand.com

1620 Préfontaine | Montréal | H1W 2N8 | Canada | Tel: +1 866 773 4246 | Fax: +1 514 221 3063

The manufacturing process of the aforementioned products is not susceptible of releasing dioxins. Additionally, the aforementioned products have not been manufactured near industrial or natural processes susceptible of releasing dioxins; nor were they manufactured using raw materials, processing aids or water resulting from such processes

Heavy Metals
Raw materials and ingredient used in the fermentation and finished product blending of Active Dry Yeast, Bacteria products, Yeast Derivatives, wine nutrients, mineral enriched yeast, and fermented soy are not listed in the EU annex or in the FDA annex for maximum levels of the heavy metals Lead (Pb), Mercury (Hg), Cadmium (Cd), and Arsenic (As). As such these regulation do not apply to Lallemand products.

Additionally the fermentation, blending and conditioning processes themselves do not bring any risk of introducing heavy metals in these products. As such, it is then safe to assume that these products can be considered not at risk for heavy metal content.

As a final reassurance of the absence of heavy metals, a monitoring program was set in place. Each year, a minimum of five (5) yeast products are tested for heavy metal content up to two (2) times per year. To this date, all products have proven to be well under the allowable limit levels.

Hazardous Substances
These products are not listed on the EU REACH CMR (Carcinogenic, Mutagenic or toxic to Reproduction) and SVHC (Substances of Very High Concern), the US NTP (National Toxicology Program), and the WHO IARC monographs. Consuming the aforementioned products represents no risk of exposure to any of the substances listed on California Proposition 65.

Tobias Fischborn
Lallemand Inc.
R&D Manager

Valid for 3 years from date of issue.

The information in this certificate has been carefully compiled to the best of our knowledge. Our products are sold subject to the understanding that prospective purchasers will conduct their own evaluations to determine the suitability of the products in their applications. Local food regulations should always be consulted with respect to specific applications and necessary declarations. Legislation may vary from country to country.

info@lallemand.com | www.lallemand.com

1620 Préfontaine | Montréal | H1W 2N8 | Canada | Tel: +1 866 773 4246 | Fax: +1 514 221 3063
