# Supplementary material for: Effect of Different Yeast Strains on Biogenic Amines, Volatile Compounds and Sensory Profile of Beer
Source: Foods. 2022 Aug 3;11(15):2317. doi: 10.3390/foods11152317 (PMC9368685; doi:10.3390/foods11152317)
Supplement: Supplementary file 1 [file foods-11-02317-s001.zip › Supplementary file 2. Beer volatile compounds profile.pdf]

Table S1. Volatile compound profile of the beer samples (A – beer fermented with *Saccharomyces cerevisiae var. Diastaticus*; B – beer fermented with *Saccharomyces cerevisiae var. bayanus*; C – beer fermented with *Brettanomyces claussenii*).

RT, min	Volatile compounds	Beer samples								
		A			B			C		
		Duration of maturation								
		1 day	30 days	90 days	1 day	30 days	90 days	1 day	30 days	90 days
34.442	Ethyl tetradecanoate	0.954 ± 0.084	0.536 ± 0.052	0.522 ± 0.05	0.615 ± 0.104	0.365 ± 0.043	0.245 ± 0.026	0.747 ± 0.139	0.32 ± 0.067	0.381 ± 0.052
38.249	Ethyl 9-hexadecenoate	0.299 ± 0.066	0.254 ± 0.024	0.56 ± 0.116	0.36 ± 0.073	0.425 ± 0.044	0.957 ± 0.176	0.203 ± 0.025	0.496 ± 0.083	0.656 ± 0.072
5.686	2,3-Butanediol	0.068 ± 0.008	0.067 ± 0.015	0.056 ± 0.01	0.088 ± 0.01	0.09 ± 0.011	0.072 ± 0.013	0.071 ± 0.014	0.075 ± 0.008	0.069 ± 0.005
8.363	3-methyl-1-Butanol acetate	nd	nd	nd	nd	nd	0.225 ± 0.035	0.501 ± 0.048	0.44 ± 0.041	0.692 ± 0.087
11.771	3-(methylthio)-1-Propanol	0.232 ± 0.039	0.182 ± 0.037	0.227 ± 0.027	0.21 ± 0.03	0.179 ± 0.021	0.235 ± 0.025	0.16 ± 0.012	0.02 ± 0.003	nd
14.857	1-Octanol	0.096 ± 0.013	0.116 ± 0.012	0.124 ± 0.018	0.042 ± 0.005	0.137 ± 0.01	0.149 ± 0.019	0.114 ± 0.015	0.152 ± 0.029	0.152 ± 0.019
16.1	2-methylbutylisovalerate	0.195 ± 0.016	0.089 ± 0.012	0.024 ± 0.003	0.241 ± 0.055	0.169 ± 0.035	0.28 ± 0.024	0.273 ± 0.035	0.19 ± 0.015	nd
17.839	4-ethylphenol	0.042 ± 0.008	0.047 ± 0.005	0.06 ± 0.013	0.069 ± 0.014	0.078 ± 0.013	0.104 ± 0.011	0.079 ± 0.01	0.081 ± 0.015	0.082 ± 0.009
18.475	Octanoic acid	0.302 ± 0.047	0.474 ± 0.038	0.536 ± 0.057	0.263 ± 0.059	0.313 ± 0.056	0.532 ± 0.043	0.317 ± 0.038	0.276 ± 0.046	0.415 ± 0.063
19.033	2-Decanol	0.153 ± 0.015	0.152 ± 0.029	0.157 ± 0.024	0.17 ± 0.024	0.187 ± 0.025	0.148 ± 0.013	0.235 ± 0.018	0.203 ± 0.03	0.211 ± 0.039
20.325	Benzeneacetic acid, ethyl ester	0.448 ± 0.042	0.433 ± 0.044	0.37 ± 0.063	0.497 ± 0.051	0.492 ± 0.064	0.468 ± 0.097	0.335 ± 0.044	0.544 ± 0.103	0.485 ± 0.073
20.651	Geraniol	0.054 ± 0.005	nd	nd	0.075 ± 0.011	nd	nd	0.192 ± 0.016	0.128 ± 0.02	0.113 ± 0.019
21.027	Nonanoic acid	0.053 ± 0.008	0.07 ± 0.012	nd	0.162 ± 0.031	0.194 ± 0.03	0.141 ± 0.018	0.058 ± 0.009	0.076 ± 0.006	nd
21.152	1-Decanol	0.051 ± 0.007	0.022 ± 0.004	nd	0.175 ± 0.017	0.137 ± 0.021	0.148 ± 0.033	0.189 ± 0.034	0.14 ± 0.022	nd
21.368	4-ethylguaiacol	0.094 ± 0.01	0.114 ± 0.018	nd	0.213 ± 0.039	0.212 ± 0.023	0.113 ± 0.024	0.124 ± 0.028	0.14 ± 0.028	nd
21.441	2-Isopropylidene-5-methylhex-4-enal	0.051 ± 0.003	nd	nd	0.104 ± 0.014	0.063 ± 0.008	0.119 ± 0.021	0.184 ± 0.033	nd	nd
21.862	Nonanoic acid, ethyl ester	0.312 ± 0.069	0.325 ± 0.063	0.359 ± 0.044	0.438 ± 0.051	0.443 ± 0.063	0.289 ± 0.043	0.264 ± 0.033	0.333 ± 0.064	0.403 ± 0.053
22.682	Geranic acid, methyl ester	0.066 ± 0.006	0.112 ± 0.021	0.109 ± 0.016	0.277 ± 0.037	0.342 ± 0.041	0.299 ± 0.026	0.367 ± 0.049	0.521 ± 0.053	0.57 ± 0.062
22.949	Acetic acid, dec-2-yl ester	0.236 ± 0.027	0.054 ± 0.01	0.044 ± 0.008	0.318 ± 0.027	0.112 ± 0.014	0.07 ± 0.012	0.245 ± 0.039	0.136 ± 0.025	0.064 ± 0.006
23.324	n-Caprylic acid isobutyl ester	0.078 ± 0.009	nd	nd	0.127 ± 0.029	0.051 ± 0.009	0.061 ± 0.007	0.17 ± 0.032	0.111 ± 0.022	nd
23.662	Ethyl decanoate	0.441 ± 0.064	0.224 ± 0.019	0.272 ± 0.036	0.208 ± 0.018	0.147 ± 0.014	0.122 ± 0.016	0.245 ± 0.026	0.12 ± 0.02	0.191 ± 0.026
23.795	Dihydro-5-pentyl-2(3H)-Furanone	0.114 ± 0.018	0.077 ± 0.017	0.08 ± 0.015	0.096 ± 0.016	0.078 ± 0.013	0.066 ± 0.006	0.083 ± 0.01	0.052 ± 0.005	0.083 ± 0.01
24.375	(Z)-6-(pent-2-enyl)tetrahydro-2H-pyran-2-one	0.101 ± 0.017	0.079 ± 0.009	0.079 ± 0.009	0.123 ± 0.02	0.107 ± 0.014	0.064 ± 0.011	0.09 ± 0.013	0.097 ± 0.012	0.05 ± 0.008
24.822	Tetradecane	0.041 ± 0.006	0.053 ± 0.009	0.066 ± 0.009	0.081 ± 0.006	0.082 ± 0.013	0.113 ± 0.026	0.063 ± 0.009	0.06 ± 0.007	0.085 ± 0.018
24.965	Diphenyl ether	0.053 ± 0.005	0.024 ± 0.004	0.026 ± 0.002	0.067 ± 0.016	0.041 ± 0.007	0.028 ± 0.006	0.061 ± 0.007	0.034 ± 0.005	nd
25.058	Acetic acid, decyl ester	0.586 ± 0.06	0.156 ± 0.02	0.088 ± 0.006	0.854 ± 0.114	0.341 ± 0.067	0.133 ± 0.019	0.697 ± 0.099	0.382 ± 0.047	0.14 ± 0.016
25.252	(-)-8-p-Menthen-2-yl, acetate, trans	0.033 ± 0.005	0.062 ± 0.008	0.055 ± 0.005	0.119 ± 0.019	0.109 ± 0.021	0.055 ± 0.008	0.185 ± 0.026	0.152 ± 0.027	0.158 ± 0.027
25.645	5-Dodecanol acetate	0.035 ± 0.003	nd	nd	0.074 ± 0.017	nd	nd	0.111 ± 0.02	0.043 ± 0.004	0.099 ± 0.02

26.028	Octanoic acid, 3-methylbutyl ester	0.183 ± 0.034	nd	nd	0.542 ± 0.073	0.685 ± 0.143	nd	0.608 ± 0.098	0.698 ± 0.094	nd
26.116	Octanoic acid, 2-methylbutyl ester	0.079 ± 0.008	0.026 ± 0.002	0.124 ± 0.011	0.132 ± 0.021	0.091 ± 0.013	0.18 ± 0.02	0.102 ± 0.02	nd	nd
27.202	Decanoic acid, propyl ester	0.06 ± 0.014	0.057 ± 0.008	0.101 ± 0.022	0.095 ± 0.019	0.092 ± 0.016	0.158 ± 0.027	0.088 ± 0.011	0.106 ± 0.017	0.423 ± 0.05
27.792	2,5-bis(1,1-dimethylethyl)phenol	0.084 ± 0.014	0.127 ± 0.01	nd	0.114 ± 0.015	0.209 ± 0.034	0.232 ± 0.025	0.174 ± 0.027	nd	nd
27.884	Butylated Hydroxytoluene	0.046 ± 0.004	0.046 ± 0.004	0.051 ± 0.009	0.057 ± 0.008	0.078 ± 0.015	0.105 ± 0.015	0.061 ± 0.009	0.075 ± 0.01	0.082 ± 0.01
28.236	δ-cadinene	0.057 ± 0.007	0.031 ± 0.003	0.055 ± 0.007	0.092 ± 0.006	0.096 ± 0.011	0.09 ± 0.007	0.059 ± 0.005	0.04 ± 0.006	0.065 ± 0.01
28.458	Eicosane	0.124 ± 0.018	nd	nd	0.087 ± 0.012	0.104 ± 0.012	nd	0.087 ± 0.01	0.04 ± 0.004	nd
29.112	(E)-Nerolidol	0.115 ± 0.013	nd	0.093 ± 0.015	0.142 ± 0.011	0.124 ± 0.013	0.144 ± 0.018	0.112 ± 0.018	0.132 ± 0.02	0.116 ± 0.02
29.84	Propanoic acid, 2-methyl-, 1-(1,1-dimethylethyl)-2-methyl-1,3-propanediyl ester	0.056 ± 0.007	0.06 ± 0.006	0.058 ± 0.005	0.062 ± 0.008	0.072 ± 0.011	0.125 ± 0.025	0.082 ± 0.01	0.098 ± 0.014	0.101 ± 0.012
30.332	Pogostol	0.558 ± 0.088	0.399 ± 0.08	0.479 ± 0.055	0.513 ± 0.037	0.449 ± 0.054	0.6 ± 0.095	0.6 ± 0.093	0.36 ± 0.057	0.436 ± 0.069
30.574	1,10-Di-epcubenol	0.04 ± 0.004	0.021 ± 0.003	nd	0.048 ± 0.006	0.055 ± 0.008	nd	0.089 ± 0.013	0.064 ± 0.008	0.083 ± 0.014
31.036	Pentadecanoic acid, 3-methylbutyl ester	0.094 ± 0.019	0.085 ± 0.005	nd	0.141 ± 0.029	0.116 ± 0.011	0.11 ± 0.022	0.213 ± 0.024	0.088 ± 0.016	nd
32.002	Cadalene	0.288 ± 0.046	0.188 ± 0.038	nd	0.197 ± 0.02	0.302 ± 0.042	0.41 ± 0.039	0.299 ± 0.049	0.426 ± 0.052	0.856 ± 0.072
34.581	Octadecane	0.156 ± 0.02	0.069 ± 0.015	0.09 ± 0.02	0.099 ± 0.015	0.072 ± 0.008	nd	0.14 ± 0.023	0.097 ± 0.009	nd
35.705	Octanoic acid, 2-phenylethyl ester	0.093 ± 0.012	0.105 ± 0.009	0.078 ± 0.014	0.141 ± 0.017	0.14 ± 0.023	0.109 ± 0.015	0.168 ± 0.026	0.168 ± 0.027	0.162 ± 0.029
35.892	Benzoic acid, tridecyl ester	0.031 ± 0.004	0.017 ± 0.002	0.073 ± 0.013	0.049 ± 0.006	0.031 ± 0.003	0.059 ± 0.005	0.033 ± 0.005	nd	0.082 ± 0.015
37.274	Hexadecanoic acid, methyl ester	0.598 ± 0.128	0.492 ± 0.076	0.578 ± 0.078	0.779 ± 0.085	0.706 ± 0.097	0.539 ± 0.066	0.721 ± 0.154	0.632 ± 0.124	0.709 ± 0.131
38.659	Hexadecanoic acid, ethyl ester	0.525 ± 0.056	nd	nd	0.755 ± 0.08	0.675 ± 0.126	nd	0.124 ± 0.02	0.123 ± 0.023	nd
9.677	methoxy-phenyl-Oxime	nd	nd	nd	nd	nd	nd	0.037 ± 0.004	0.017 ± 0.003	nd
12.033	Hexanoic acid	nd	nd	nd	nd	nd	nd	nd	nd	0.291 ± 0.045
12.932	Acetic acid, hexyl ester	0.017 ± 0.001	nd	nd	0.02 ± 0.002	nd	nd	0.033 ± 0.007	0.02 ± 0.003	nd
15.558	2-Nonanone	0.057 ± 0.007	0.029 ± 0.003	0.024 ± 0.003	0.038 ± 0.004	0.026 ± 0.003	nd	0.034 ± 0.006	0.012 ± 0.002	nd
15.825	β-linalool	nd	nd	nd	nd	nd	0.149 ± 0.026	nd	nd	nd
16.274	Phenylethyl Alcohol	nd	nd	nd	nd	0.024 ± 0.004	nd	nd	nd	nd
17.756	Ethyl octanoate	0.033 ± 0.005	0.025 ± 0.005	0.023 ± 0.004	0.046 ± 0.008	0.03 ± 0.003	nd	0.066 ± 0.011	0.035 ± 0.003	nd
18.978	Octanoic acid, ethyl ester	0.022 ± 0.003	nd	nd	0.069 ± 0.004	nd	nd	0.124 ± 0.022	0.059 ± 0.004	nd
19.866	Citronellol	0.014 ± 0.002	nd	nd	0.01 ± 0.001	0.038 ± 0.006	nd	0.027 ± 0.003	0.008 ± 0.001	nd
20.958	2-Undecanol	nd	nd	nd	nd	nd	nd	0.053 ± 0.009	nd	nd
21.79	2-Undecanone	nd	nd	nd	0.079 ± 0.014	nd	nd	0.079 ± 0.008	0.086 ± 0.015	nd
22.01	Methyl nonyl carbinol	nd	nd	0.021 ± 0.003	nd	0.016 ± 0.002	nd	nd	0.029 ± 0.005	0.065 ± 0.011
22.125	1H-2-Indenone,2,4,5,6,7,7a-hexahydro-3-(1-methylethyl)-7a-methyl	0.057 ± 0.005	0.028 ± 0.004	nd	0.022 ± 0.003	0.023 ± 0.005	nd	0.029 ± 0.004	0.02 ± 0.002	nd

22.279	Acetic acid, nonyl ester	0.027 ± 0.005	0.06 ± 0.007	nd	0.046 ± 0.006	nd	nd	nd	nd	nd
22.428	4-vinylguaiacol	0.025 ± 0.006	0.045 ± 0.005	0.031 ± 0.005	0.027 ± 0.005	0.087 ± 0.017	nd	0.024 ± 0.002	0.09 ± 0.009	nd
23.446	Citronellol acetate	nd	nd	nd	0.069 ± 0.005	nd	nd	0.081 ± 0.018	nd	nd
24.086	n-Decanoic acid	nd	nd	nd	nd	0.03 ± 0.004	0.046 ± 0.007	0.057 ± 0.007	nd	nd
24.745	Decanoic acid, ethyl ester	0.007 ± 0.001	0.009 ± 0.002	nd	0.01 ± 0.002	0.011 ± 0.002	nd	0.007 ± 0.001	0.011 ± 0.001	nd
25.301	Florhydral	0.02 ± 0.004	nd	nd	0.029 ± 0.005	nd	nd	0.048 ± 0.008	0.015 ± 0.002	nd
26.268	3-methoxy-5-methyl-2-Cyclopenten-1-one	0.083 ± 0.014	nd	nd	0.105 ± 0.015	nd	nd	0.102 ± 0.013	nd	nd
26.355	(E)-β-Farnesene	0.052 ± 0.005	nd	nd	0.084 ± 0.011	nd	nd	0.054 ± 0.011	nd	nd
26.766	Dodecanol	0.049 ± 0.007	nd	nd	0.058 ± 0.011	0.022 ± 0.004	nd	0.055 ± 0.011	0.034 ± 0.003	nd
27.026	γ-Muurolene	nd	nd	nd	nd	nd	nd	0.033 ± 0.005	nd	nd
27.297	Undecanoic acid, ethyl ester	nd	nd	nd	0.017 ± 0.002	nd	nd	nd	nd	nd
27.341	Viridiflorol	0.029 ± 0.006	nd	nd	0.032 ± 0.004	nd	nd	0.026 ± 0.004	0.038 ± 0.005	nd
27.51	2-Tridecanol	0.049 ± 0.009	0.023 ± 0.004	nd	0.06 ± 0.01	0.038 ± 0.005	nd	0.054 ± 0.011	0.047 ± 0.009	nd
27.572	α-Selinene	0.012 ± 0.003	0.022 ± 0.003	nd	0.011 ± 0.002	0.023 ± 0.004	nd	0.014 ± 0.003	0.026 ± 0.005	nd
27.727	cis-1,2-Cyclododecanediol	0.086 ± 0.005	nd	nd	0.098 ± 0.007	nd	nd	0.085 ± 0.016	nd	nd
28.021	γ-cadinene	0.09 ± 0.017	nd	nd	0.029 ± 0.002	0.056 ± 0.011	nd	0.14 ± 0.02	nd	nd
28.767	α-Calacorene	nd	nd	0.297 ± 0.068	nd	nd	nd	nd	nd	nd
29.034	Dodecanoic acid	nd	nd	nd	nd	nd	nd	0.033 ± 0.005	0.142 ± 0.028	nd
29.449	Palustrol	0.02 ± 0.004	nd	nd	0.014 ± 0.002	nd	nd	nd	nd	nd
30.17	Lauryl acetate	0.04 ± 0.008	0.018 ± 0.004	nd	nd	nd	nd	nd	nd	nd
30.214	(Z)-α-Bisabolene	0.023 ± 0.002	0.018 ± 0.002	0.024 ± 0.002	0.02 ± 0.003	0.022 ± 0.003	nd	0.03 ± 0.005	0.009 ± 0.002	nd
30.483	Humulene epoxide II	0.03 ± 0.005	nd	0.041 ± 0.004	0.03 ± 0.002	0.04 ± 0.004	0.052 ± 0.008	0.029 ± 0.003	0.025 ± 0.002	nd
30.89	Epicubenol	0.052 ± 0.007	0.023 ± 0.004	nd	0.015 ± 0.003	0.023 ± 0.002	nd	0.017 ± 0.002	0.014 ± 0.002	nd
31.174	epi-α-Cadinol	0.029 ± 0.005	0.023 ± 0.003	nd	0.042 ± 0.008	0.031 ± 0.002	nd	0.048 ± 0.004	nd	nd
31.795	1-Tetradecanol	0.022 ± 0.002	nd	nd	0.012 ± 0.002	nd	nd	0.014 ± 0.002	nd	nd
32.914	(E,E)-Farnesol	nd	nd	nd	0.014 ± 0.002	nd	nd	0.018 ± 0.003	0.022 ± 0.002	nd
33.344	Isobutyl laurate	nd	nd	nd	nd	nd	nd	nd	0.132 ± 0.011	nd
35.438	Benzoic acid, hexyl ester	0.016 ± 0.002	nd	nd	nd	nd	nd	nd	nd	nd
35.55	Isoamyl laurate	nd	nd	nd	nd	nd	nd	nd	0.071 ± 0.011	nd
